# Inactivation of *Zeb1* in GRHL2-deficient mouse embryos rescues mid-gestation viability and secondary palate closure

**DOI:** 10.1242/dmm.042218

**Published:** 2020-03-25

**Authors:** Marina R. Carpinelli, Michael E. de Vries, Alana Auden, Tariq Butt, Zihao Deng, Darren D. Partridge, Lee B. Miles, Smitha R. Georgy, Jody J. Haigh, Charbel Darido, Simone Brabletz, Thomas Brabletz, Marc P. Stemmler, Sebastian Dworkin, Stephen M. Jane

**Affiliations:** 1 Department of Medicine, Central Clinical School, Monash University, 99 Commercial Road, Melbourne, VIC 3004, Australia; 2 Australian Centre for Blood Diseases, Central Clinical School, Monash University, 99 Commercial Road, Melbourne, VIC 3004, Australia; 3 Department of Experimental Medicine I, Nikolaus-Fiebiger Center for Molecular Medicine, Friedrich-Alexander University of Erlangen-Nürnberg, Erlangen 91054, Germany

**Keywords:** Grhl2, Zeb1, Palate, EMT, Explant

## Abstract

Cleft lip and palate are common birth defects resulting from failure of the facial processes to fuse during development. The mammalian grainyhead-like (*Grhl1-3*) genes play key roles in a number of tissue fusion processes including neurulation, epidermal wound healing and eyelid fusion. One family member, *Grhl2*, is expressed in the epithelial lining of the first pharyngeal arch in mice at embryonic day (E)10.5, prompting analysis of the role of this factor in palatogenesis. *Grhl2*-null mice die at E11.5 with neural tube defects and a cleft face phenotype, precluding analysis of palatal fusion at a later stage of development. However, in the first pharyngeal arch of *Grhl2*-null embryos, dysregulation of transcription factors that drive epithelial-mesenchymal transition (EMT) occurs. The aberrant expression of these genes is associated with a shift in RNA-splicing patterns that favours the generation of mesenchymal isoforms of numerous regulators. Driving the EMT perturbation is loss of expression of the EMT-suppressing transcription factors *Ovol1* and *Ovol2*, which are direct GRHL2 targets. The expression of the miR-200 family of microRNAs, also GRHL2 targets, is similarly reduced, resulting in a 56-fold upregulation of *Zeb1* expression, a major driver of mesenchymal cellular identity. The critical role of GRHL2 in mediating cleft palate in *Zeb1^−/−^* mice is evident, with rescue of both palatal and facial fusion seen in *Grhl2^−/−^;Zeb1^−/−^* embryos. These findings highlight the delicate balance between GRHL2/ZEB1 and epithelial/mesenchymal cellular identity that is essential for normal closure of the palate and face. Perturbation of this pathway may underlie cleft palate in some patients.

## Abstract (precis)

**Summary:** Epithelial transcription factor GRHL2 is required for face closure while mesenchymal transcription factor ZEB1 is required for palate closure. Surprisingly, animals lacking both factors close their face and secondary palate.

## INTRODUCTION

Cleft lip and/or palate affects approximately 1 in 700 live births (Dixon et al., 2011) and is associated with significant morbidity. This birth defect arises when facial processes fail to fuse during embryonic development. The upper lip and anterior region of the palate, termed primary palate, has a separate ontogeny from the posterior region, termed secondary palate. The upper lip and primary palate are derived from fusion between the medial nasal processes (MNPs) and maxillary processes (MXPs). The secondary palate is formed when palatal shelves outgrow from the MXPs, initially downwards adjacent to the tongue, then reorientating to grow towards the midline. Fusion between the palatal shelves creates a midline seam of epithelium that dissolves to allow mesenchymal confluence. In the mouse, MXP-MNP fusion occurs at embryonic day (E)10.5, while palatal shelf outgrowth and fusion occur between E11.5 and E15.5, resulting in palate formation being complete by E17 (Bush and Jiang, 2012). In the sixth week of human gestation, the MXPs, MNPs and lateral nasal process (LNP) fuse, and the palatal shelves begin to grow from the oral side of the MXPs. In the eighth week, the palatal shelves elevate above the tongue and merge in the midline. They also fuse with primary palate and nasal septum, with fusion complete by the twelfth week of gestation (Lan et al., 2015).

Numerous genes have been implicated in the regulation of mammalian palatogenesis (Bush and Jiang, 2012), including the grainyhead-like 3 (*GRHL3*) gene (Peyrard-Janvid et al., 2014), a member of a large family of highly conserved developmental transcription factors (Wilanowski et al., 2002). The first-discovered member of this family, *Drosophila* grainyhead (*grh*), is required for formation of the head skeleton, dorsal hole closure, integrity of the cuticle, and other cellular polarity and migration events during fly development (Bray and Kafatos, 1991). Truncating mutations in human *GRHL3* cause cleft palate (Mangold et al., 2016), while a missense variant is associated with increased risk of cleft palate at the population level (Leslie et al., 2016). The congenital disorder Van Der Woude syndrome, involving cleft lip and/or palate with lower lip pits, can also be caused by dominant mutations in *GRHL3* (Peyrard-Janvid et al., 2014). *Grhl3* is also critical for neural tube closure, a role it shares with its nearest mammalian paralog, *Grhl2*. We and others have shown that *Grhl2^−/−^* mouse embryos exhibit a cleft face, cranioschisis and an open posterior neuropore at E10.5 (Rifat et al., 2010; Werth et al., 2010). Embryos carrying N-ethyl-N-nitrosourea (ENU)-induced mutations in *Grhl2* can survive until advanced stages of development, at which point they display a cleft upper jaw (Menke et al., 2015; Pyrgaki et al., 2011). The non-neural ectoderm adjacent to the neural plate displays mesenchymal characteristics in *Grhl2* mutant embryos, including aberrant vimentin expression and increased cell motility (Ray and Niswander, 2016). The resulting lack of epithelial integrity prevents apposition of the neural folds, resulting in failed neural tube closure.

ZEB1 is another transcription factor implicated in palatogenesis. *Zeb1^−/−^* mouse embryos die neonatally with a cleft palate and widespread skeletal abnormalities (Takagi et al., 1998). *Zeb1* is expressed in the mesenchyme but not the epithelium during palatal shelf outgrowth (Liu et al., 2008; Shin et al., 2012). *Zeb1^−/−^* palatal mesenchyme fails to express the mesenchymal marker vimentin but ectopically expresses the epithelial marker E-cadherin at E16.5 (Liu et al., 2016). A spontaneously arising mutant, the Twirler mouse, displays cleft lip and secondary palate associated with failure of palatal shelf outgrowth (Gong et al., 2000). This phenotype is due to a point mutation in intron 1 of *Zeb1* that dysregulates its expression (Kurima et al., 2011). Although mutations in *ZEB1* are not associated with cleft palate in humans, haploinsufficiency causes posterior polymorphic corneal dystrophy, a characteristic feature of which is acquisition of epithelial characteristics by corneal endothelium (Krafchak et al., 2005; Liskova et al., 2016).

The developing palate is composed of epithelial and mesenchymal cell types. Epithelial cells are immotile and characterized by their apical-basal polarity, attachment to a basal lamina, cortical ring of actin and expression of intercellular tight and adherens junctions. In contrast, mesenchymal cells exhibit anteroposterior polarity and actin stress fibres, are not attached to a basal lamina and can be motile. The epithelial-mesenchymal transition (EMT) is a phenotype shift that occurs during embryonic development and cancer metastasis. Where a cell sits on the epithelial-mesenchymal phenotypic spectrum at any given time is determined by expression of microRNAs, transcription factors and splicing regulators (Nieto et al., 2016). The miR-200 and miR-34 family microRNAs, GRHL2, OVOL1 and OVOL2 transcription factors, and ESRP1 and ESRP2 splicing regulators promote epithelial cellular identity. The ZEB1, ZEB2, SNAI1, PRRX1 and TWIST1 transcription factors and the QKI, SRSF1 and RBFOX2 splicing factors promote mesenchymal cellular identity. A number of observations link GRHL2 to regulation of EMT, particularly the strong promotion and maintenance of epithelial identity, albeit largely in non-developmental contexts. GRHL2 suppresses EMT in a breast cancer cell line by directly repressing the *ZEB1* promoter (Cieply et al., 2013, 2012), while key EMT suppressors are directly transactivated by GRHL2, including *MIR200B*, *MIR200A*, *MIR429* (Chung et al., 2016) and *Ovol2* (Aue et al., 2015).

In this paper, we demonstrate that GRHL2 maintains the cellular identity of palatal epithelium by transactivating EMT-suppressing microRNAs, transcription factors and splicing regulators. These findings indicate that the pathway by which GRHL2 suppresses EMT in cancer is also crucial for palatogenesis.

## RESULTS

### The MXPs of *Grhl2^−/−^* embryos are smaller than those of wild-type littermates at E10.5

**Fig. 1.**
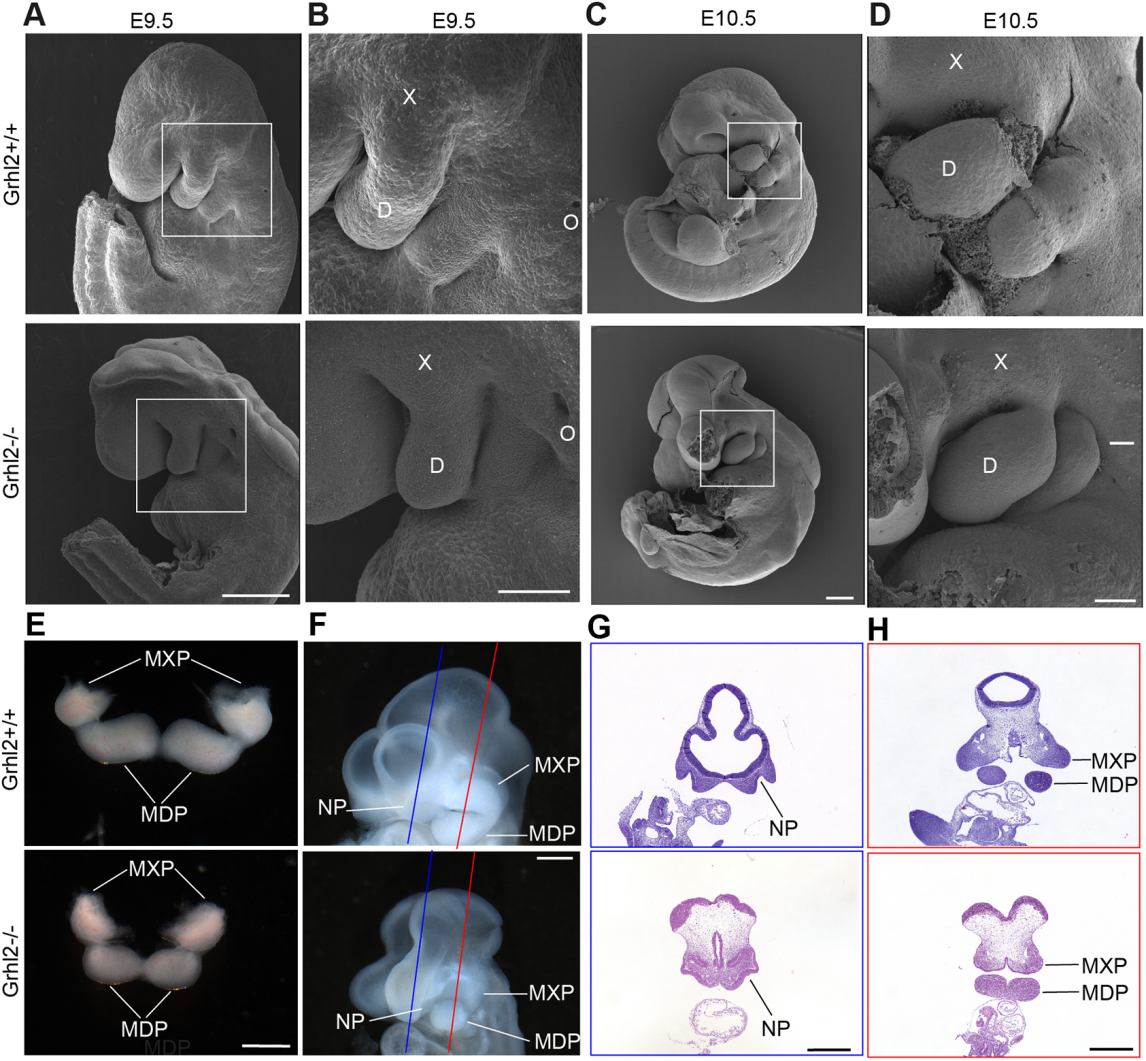
***Grhl2^−/−^* embryos have small maxillary processes.** (A-D) Scanning electron micrographs of *Grhl2^+/+^* and *Grhl2^−/−^* E9.5 (A,B) and E10.5 (C,D) embryos. Boxes indicate the location of adjacent higher-magnification images. D, mandibular process; O, otic pit; X, maxillary process. (E) PA1 dissected from E10.5 embryos. (F) Planes of sections through E10.5 embryos shown in G (blue line) and H (red line). (G,H) Coronal sections through the nasal process (G) and maxillary process (H) stained with Haematoxylin and Eosin (H&E). MDP, mandibular process; MXP, maxillary process; NP, nasal pit. Images are representative of four embryos of each genotype. Scale bars: 250 μm (A,C), 100 μm (B,D) and 500 μm (E-H).

*Grhl2* is expressed in the epithelium lining the maxillary and nasal processes at E10.5 (Brouns et al., 2011), with expression continuing in oral epithelium until E17.5 (Auden et al., 2006). This expression pattern is consistent with *Grhl2* playing a role in closure of the palate. As *Grhl2^−/−^* embryos have a cleft face, we first determined whether their maxillary and nasal processes formed normally between E9.5 and E10.5. Scanning electron micrographs revealed that the MXPs were abnormally small in *Grhl2^−/−^* embryos at E10.5 (Fig. 1A-D). The mean ratio of MXP/MDP area was 0.96 for wild-type embryos and 0.78 for *Grhl2^−/−^* embryos, although this difference was not statistically significant. Next, we dissected the first pharyngeal arch (PA1) at E10.5, and noted again that the *Grhl2^−/−^* MXPs were reduced in size (Fig. 1E). Images of the head of E10.5 embryos revealed that the nasal processes were malformed in *Grhl2^−/−^* embryos, making it infeasible to determine whether the lambdoid junction had formed (Fig. 1F). We cut defined section planes through the nasal and maxillary processes of E10.5 embryos and determined that the nasal pits were present in *Grhl2^−/−^* embryos, as were the MXPs, which stained darkly with Haematoxylin and Eosin (H&E) (Fig. 1G,H). These findings showed that *Grhl2^−/−^* embryos have small MXPs at E10.5. In order to determine whether *Grhl2^−/−^* embryos had a deficiency of cranial neural crest cells, we stained coronal sections through E10.5 PA1 for SOX9. *Grhl2^−/−^* embryos exhibited normal numbers of SOX9-positive neural crest cells in their MXP and mandibular process (MDP) mesenchyme at E10.5 (Fig. S1A,B). In order to determine whether GRHL2 is required in neural crest cells for palate closure, we conditionally inactivated *Grhl2* using *Wnt1Cre* (Danielian et al., 1998). *Grhl2^+/−^;Wnt1Cre^+^*×*Grhl2^fl/fl^* timed matings were performed and the embryos harvested at E17.5. Of 55 embryos harvested, 16 (29%) were *Grhl2^fl/−^;Wnt1Cre^+^*. This was not significantly different from the Mendelian expectation of 25%, indicating that embryos lacking *Grhl2* in neural crest cells were viable to E17.5. Furthermore, skeletal preparations stained with Alizarin Red and Alcian Blue revealed normal palate closure in *Grhl2^fl/−^;Wnt1Cre^+^* conditional knockout embryos at E17.5 (Fig. S1C). These findings indicate that GRHL2 is not required for neural crest cells to populate the first pharyngeal arch or required in neural crest cells for palate closure.

### *Grhl2^−/−^* first pharyngeal arch epithelium displays mesenchymal characteristics

**Fig. 2.**
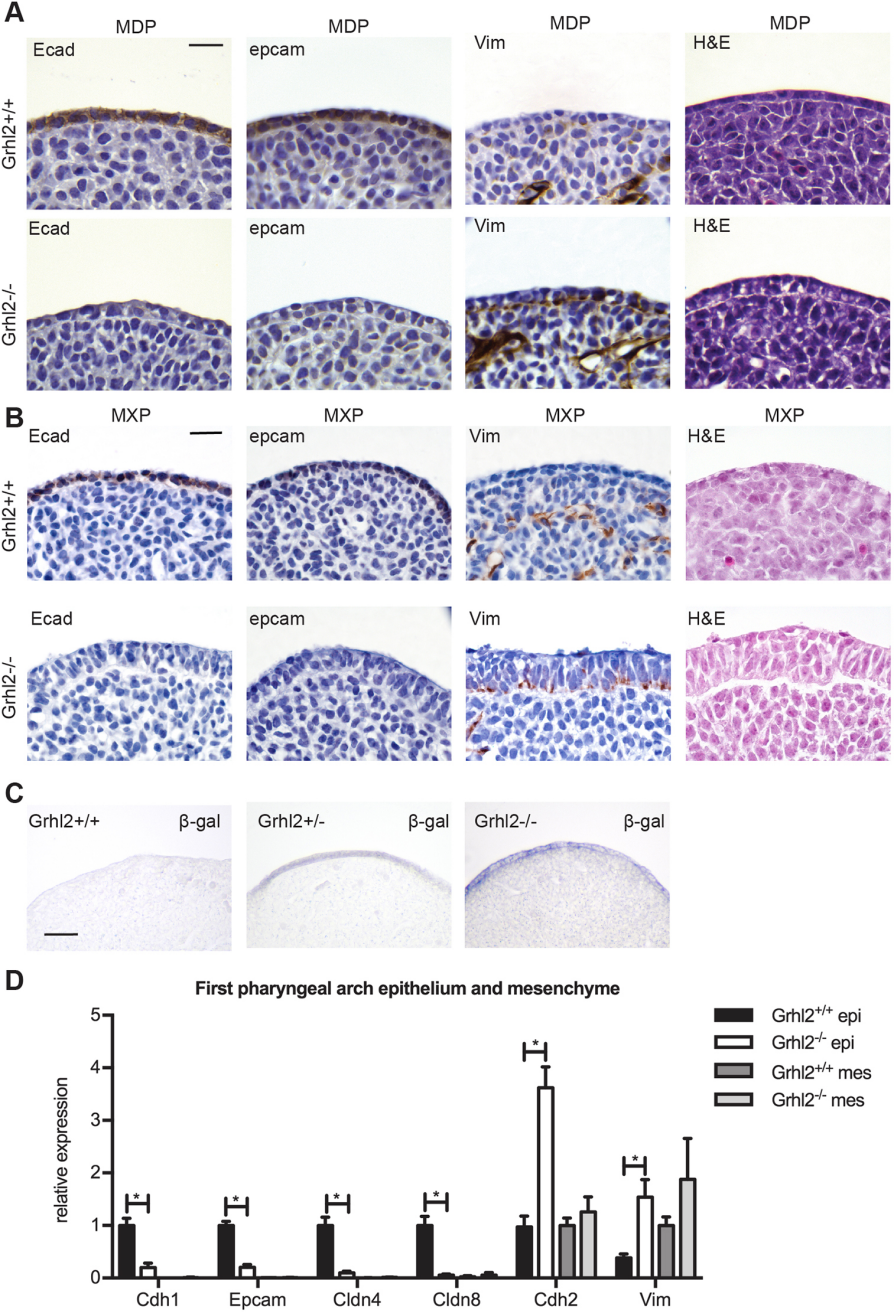
***Grhl2^−/−^* PA1 epithelium has mesenchymal characteristics.** (A) Transverse sections through the E10.5 PA1 MDP stained by immunohistochemistry for E-cadherin (Ecad), Epcam or vimentin (Vim), or with H&E. Images are representative of four embryos. (B) As for panel A, except images are of coronal sections through the MXP. (C) Transverse sections through the MDP of E10.5 embryos stained for β-galactosidase (β-gal) activity. (D) Q-RT-PCR on E10.5 PA1 epithelium (epi) and mesenchyme (mes). Graph shows mean±s.d. *n*=6 embryos. *Grhl2^+/+^* and *Grhl2^−/−^* epithelium were compared using Student's *t*-tests corrected for multiple comparisons with the Holm-Sidak method. *P<0.0001. Scale bars: 20 μm (A,B) and 40 μm (C).

As GRHL2 is required to maintain the epithelial integrity of non-neural ectoderm at E8.5 (Ray and Niswander, 2016), we determined whether absence of this transcription factor perturbs E-cadherin and vimentin expression at E10.5. The first pharyngeal arch (PA1) comprises the MXPs, which form the palate, and the MDPs, which form the lower jaw. We collected sections through the PA1 of *Grhl2^+/+^* and *Grhl2^−/−^* embryos at E10.5, and performed immunohistochemical staining on the MDPs and MXPs (Fig. 2A,B). The epithelial markers E-cadherin and Epcam were downregulated in *Grhl2^−/−^* epithelium. Conversely, the mesenchymal marker vimentin was ectopically expressed in *Grhl2^−/−^* epithelium in this system. Staining for β-galactosidase (which reports expression from the *Grhl2* gene-targeted allele) confirmed that *Grhl2* was expressed in the epithelial but not mesenchymal compartments of the PA1 (Fig. 2C). We isolated the epithelial and mesenchymal compartments of PA1 and performed quantitative reverse transcription PCR (Q-RT-PCR) using primers for epithelial (*Cdh1*, *Epcam*, *Cldn4*, *Cldn8*) and mesenchymal (*Cdh2*, *Vim*) gene transcripts. Epithelial genes were downregulated and mesenchymal genes were upregulated in *Grhl2^−/−^* PA1 epithelium compared to that of wild-type controls (Fig. 2D). In contrast, expression of these genes was unaltered in *Grhl2^−/−^* PA1 mesenchyme, in keeping with the lack of *Grhl2* expression in this tissue in wild-type mice.

**Fig. 3.**
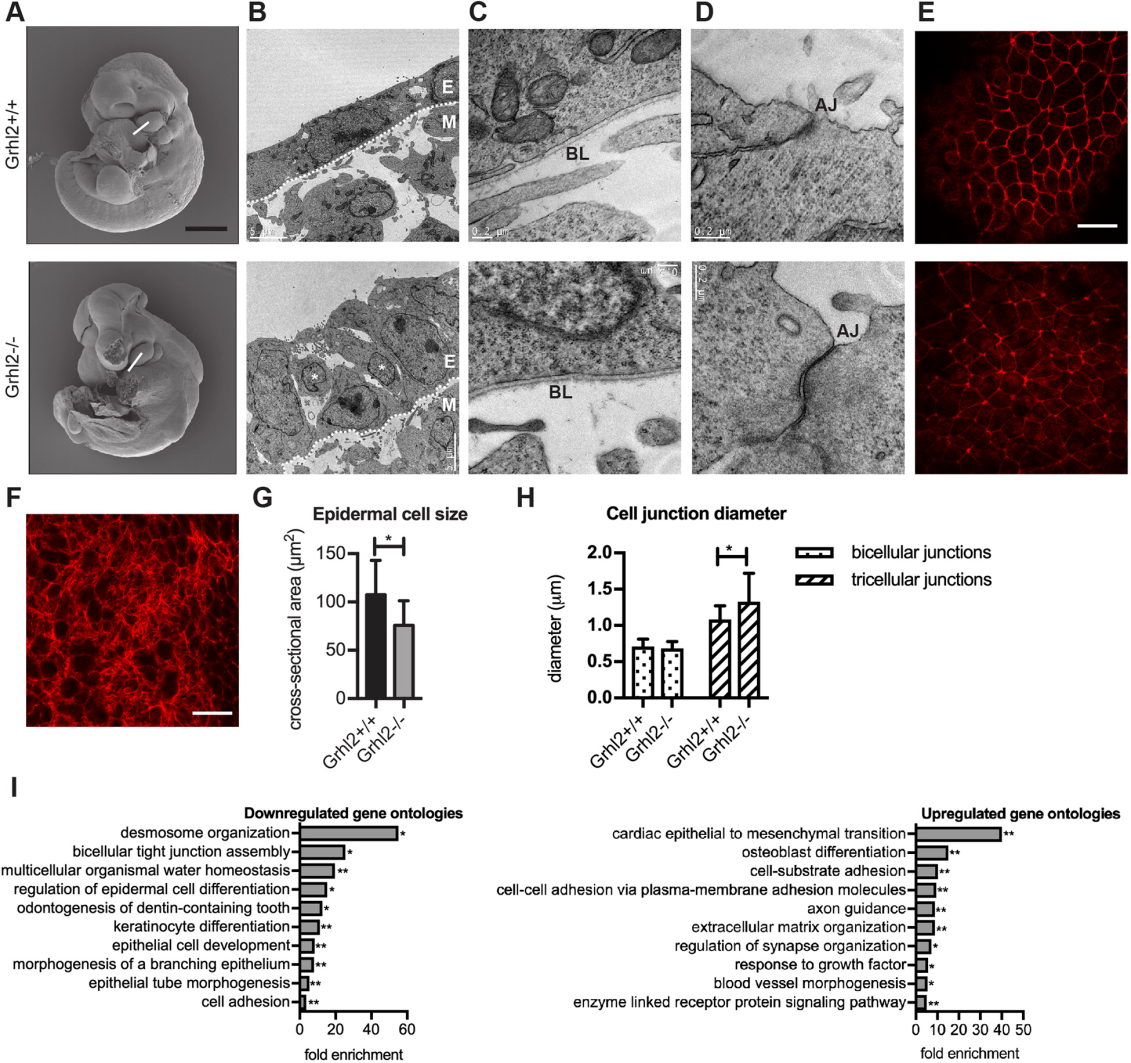
***Grhl2^-/-^* epidermis retains some epithelial characteristics.** (A) Scanning electron microscopy (SEM) images of E10.5 *Grhl2^+/+^* and *Grhl2^-/-^* embryos. Images are repeated from Fig. 1C to illustrate the plane of sections used for transmission electron microscopy (TEM; white line). (B) 5000× TEM image of *Grhl2^−/−^* PA1 epithelium (E) and underlying mesenchyme (M). Asterisks indicate epithelial cells not contacting the basal lamina (dotted line). (C) 70,000× TEM image of the basal end of a PA1 epithelial cell. BL, basal lamina. (D) 120,000× TEM image of the apical end of a PA1 epithelial cell. AJ, apical junction. (E) Confocal microscopy slice through MDP epithelium after staining with rhodamine-phalloidin. Red staining shows the cortical actin ring that surrounds each epithelial cell. (F) An orthoslice through the mesenchymal region of a MDP showing actin stress fibres. SEM, TEM and confocal images are representative of four embryos. (G) PA1 epithelial cell size measured from images in E and similar images of three other *Grhl2^+/+^* and three other *Grhl2^−/−^* embryos. Cross-sectional area of 20 MDP epithelial cells was measured in each embryo. *P<0.0001 using Mann–Whitney test. (H) PA1 epithelial junction diameter measured from images similar to those in E. 110-163 bicellular junctions and 96-104 tricellular junctions were measured. *P<0.0001 using Mann–Whitney test. (I) Most strongly downregulated and upregulated gene ontologies in *Grhl2^−/−^* PA1. *P<0.05, **P<0.01 using binomial test with Bonferroni correction. Graphs in G and H show mean±s.d. Scale bars: 500 μm (A), 5 μm (B), 0.2 μm (C,D) and 20 μm (E,F).

To determine which epithelial cellular characteristics were retained in the absence of GRHL2, we imaged the PA1 of E10.5 embryos using cryo-electron microscopy. Images through the MDP revealed that the wild-type arch was lined by an epithelium of single-cell thickness, with all cells in contact with the basal lamina (Fig. 3A,B). In contrast, the *Grhl2^−/−^* epithelium was thicker and presented with a disorderly cellular arrangement, with some cells not contacting the basal lamina, characteristic of ‘mesenchymal’ cell behaviour. Higher-magnification images revealed that the basal lamina was intact in *Grhl2^−/−^* embryos (Fig. 3C), and apical junctions were readily identifiable at the epithelial surface (Fig. 3D), indicating some maintenance of apico-basal polarity. Confocal microscope optical slices at various depths through the MDPs of embryos stained for filamentous actin revealed that cortical actin was retained in *Grhl2^−/−^* surface epithelium (Fig. 3E) but was not apparent in the underlying mesenchyme (Fig. 3F). Furthermore, the cortical area of the *Grhl2^−/−^* epithelial cells was reduced (Fig. 3G). Cortical actin width was normal at bicellular junctions but increased at tricellular junctions in *Grhl2^−/−^* epithelium (Fig. 3H). These data suggest that *Grhl2^−/−^* PA1 epithelium has a cellular phenotype intermediate between that of wild-type epithelium and mesenchyme.

To further examine the shift towards mesenchymal gene expression in the *Grhl2^−/−^* epithelium, we performed RNA sequencing (RNAseq) on the dissected PA1 at E10.5. We identified 163 genes that were downregulated (Table S1) and 117 genes that were upregulated (Table S2) in *Grhl2^−/−^* PA1 compared to wild-type PA1. Given that the PA1 is composed of epithelial and mesenchymal cells, we expect some of the observed gene expression changes to have arisen from each compartment. We next searched for gene ontologies that were enriched in the downregulated and upregulated gene lists. The gene ontologies most strongly down-regulated in the absence of GRHL2 were integral to epithelial functions, including ‘desmosome organization’, ‘bicellular tight junction assembly’, ‘multicellular organismal water homeostasis’ and ‘regulation of epidermal cell differentiation’ (Fig. 3I). In contrast, the most significantly upregulated gene ontology in *Grhl2^−/−^* PA1 was ‘cardiac epithelial to mesenchymal transition’, due to upregulation of *Has2*, *Tmem100*, *Tgfbr3*, *Bmp2* and *Hey1*. Remodelling of the primitive heart into a mature, four-chambered heart requires the formation of endocardial cushion tissue in the atrioventricular canal and outflow tract. Endocardial cells undergo EMT to delaminate and repopulate underlying extracellular matrix to form the cushion tissues. HAS2 synthesizes the extracellular matrix component hyaluronan, required for endocardial cell EMT (Camenisch et al., 2000). TMEM100 is a transmembrane protein and HEY1 is a transcription factor also required for endocardial cell EMT (Fischer et al., 2007; Mizuta et al., 2015). BMP2 binds to the TGFBR3 cell surface receptor to mediate EMT in chick endocardial cushion explants (Kirkbride et al., 2008). This indicated that a widespread shift in gene expression from epithelial to mesenchymal occurs in the absence of GRHL2. Table S3 lists genes from key ontologies that were downregulated in *Grhl2^−/−^* PA1. Table S4 lists genes from key ontologies that were upregulated in *Grhl2^−/−^* PA1. We next performed gene set enrichment analysis to determine whether defined sets of genes showed significant, concordant differences between wild-type and *Grhl2^−/−^* PA1 (Subramanian et al., 2005). By interrogating the Molecular Signatures Database (http://software.broadinstitute.org/gsea/msigdb/index.jsp), we found a significant correlation with a set of epithelial genes that are repressed by ZEB1 (Aigner et al., 2007) (Fig. S2). These genes were upregulated by knockdown of *ZEB1* in a metastatic breast cancer cell line and are thus likely to be targets of ZEB1-mediated transcriptional repression. Of the 26 mouse orthologues, 17 displayed core enrichment, meaning that this subset of genes contributes most to the enrichment result. Furthermore, 11 of these 26 mouse orthologues were significantly downregulated in *Grhl2^−/−^* PA1 compared to wild-type PA1, indicating a strong inverse correlation between these datasets.

### EMT transcription factors, microRNAs and splicing patterns are dysregulated in the epithelial lining of the first pharyngeal arch in *Grhl2^−/−^* embryos

**Fig. 4.**
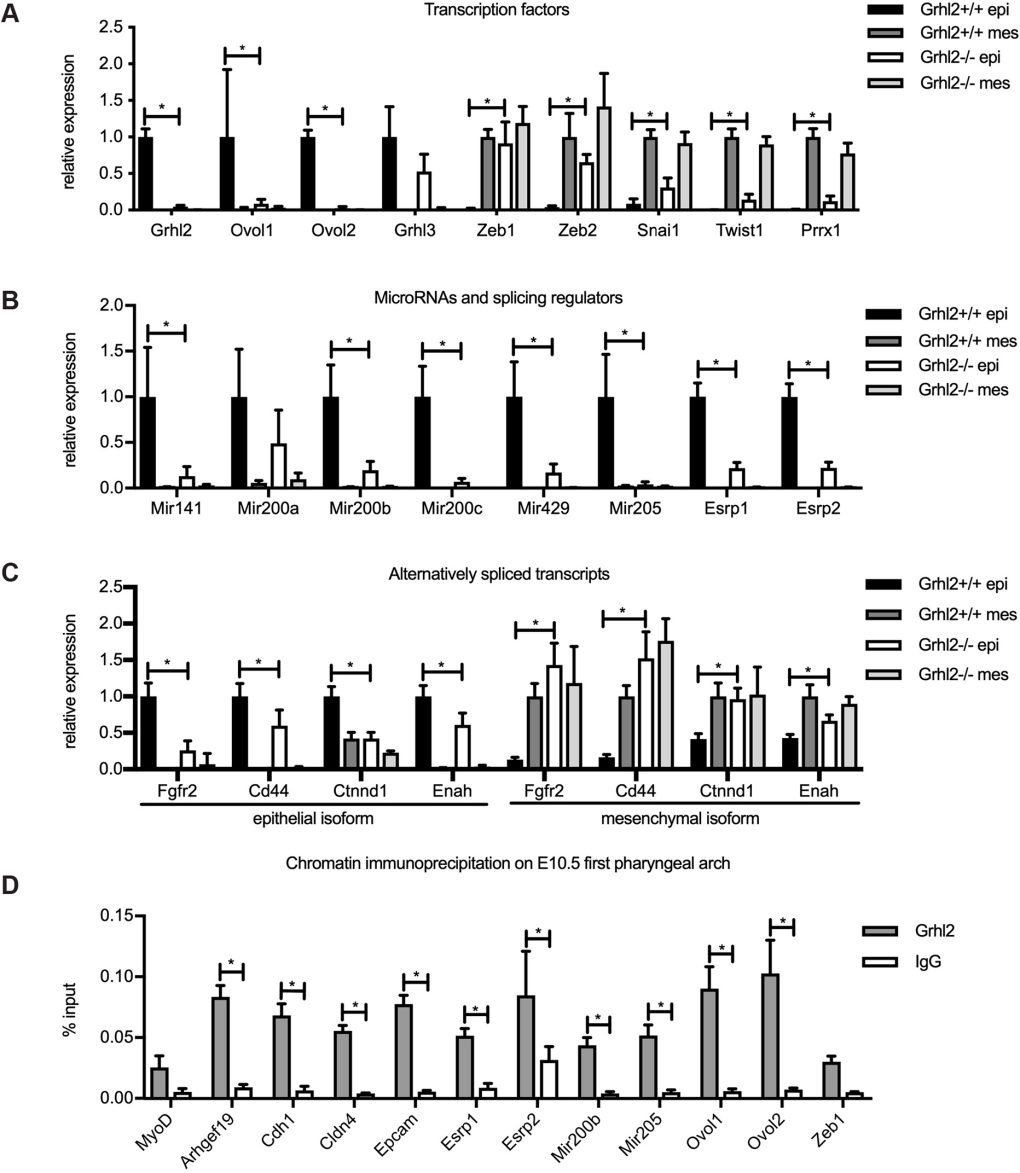
**GRHL2 maintains epithelial cellular identity via multiple direct target genes.** Q-RT-PCR on E10.5 PA1 epithelium (epi) and mesenchyme (mes). (A) Epithelial and mesenchymal transcription factors. (B) *Zeb1*-repressing microRNAs and epithelial splicing regulators. (C) Isoform-specific Q-RT-PCR for *Fgfr2*, *Cd44*, *Ctnnd1* and *Enah*. *Grhl2^+/+^* and *Grhl2^−/−^* epithelium were compared using Student's *t*-tests corrected for multiple comparisons with the Holm-Sidak method. *P<0.05. *n*=5-6 embryos. (D) ChIP on E10.5 PA1 with Grhl2 antibody or normal rabbit immunoglobulin (IgG) antibody, followed by PCR with primers spanning predicted GRHL2 binding sites. Results are representative of three independent experiments. *P<0.0001 by two-way ANOVA with Sidak's multiple comparison test. Graphs show mean±s.d of quadruplicate Q-RT-PCRs.

To determine the mechanism underpinning the shift towards mesenchymal phenotype in the absence of GRHL2, we quantified the expression of the transcription factors and microRNAs that regulate EMT. We performed Q-RT-PCR on PA1 epithelium and mesenchyme of *Grhl2^−/−^* and wild-type embryos at E10.5. This revealed that the *Ovol1* and *Ovol2* transcriptional repressors, which suppress EMT, were downregulated, whereas the *Zeb1*, *Zeb2*, *Snai1*, *Twist1* and *Prrx1* master regulators, which promote EMT, were upregulated (Fig. 4A). Of note, *Zeb1* and *Zeb2* were expressed at equivalent levels in *Grhl2^−/−^* epithelium as in wild-type mesenchyme, implying that they were completely dysregulated in the absence of GRHL2. In contrast, although *Snai1*, *Twist1* and *Prrx1* were also upregulated, they were still expressed at submesenchymal levels. Interestingly, of these genes, only the epithelial transcription factors were detected as differentially expressed in RNAseq (Table S1). In that dataset, *Grhl2* was downregulated 8-fold, *Ovol1* was downregulated 14-fold and *Ovol2* was downregulated 16-fold in *Grhl2^−/−^* PA1 compared to wild-type PA1. Likely, we failed to detect misexpression of mesenchymal transcription factors in the RNAseq experiment because the bulk of PA1 is composed of mesenchyme, and hence the ectopic expression of these factors in epithelium did not significantly increase their overall expression level. Because of its known role in palate closure, we also measured the expression of *Grhl3*. This transcription factor displayed epithelial-specific expression but was not significantly downregulated in *Grhl2^−/−^* PA1 epithelium. Similarly, this factor was not misexpressed in the RNAseq dataset. As miR-200 microRNAs and miR-205 inhibit the expression of *Zeb1* and *Zeb2* (Gregory et al., 2008), we postulated that these might be downregulated in *Grhl2^−/−^* epithelium. Measurement of these microRNAs by Q-RT-PCR confirmed this, with miR-200 family microRNAs *Mir141*, *Mir200b*, *Mir200c* and *Mir429*, along with *Mir205*, significantly downregulated in *Grhl2^−/−^* PA1 epithelium compared to wild-type PA1 (Fig. 4B). MicroRNAs were not captured in the RNAseq experiment.

As patterns of pre-mRNA splicing change during EMT (Nieto et al., 2016), we next measured expression of the epithelial splicing regulator genes *Esrp1* and *Esrp2*. These factors promote splicing into the epithelial isoform over the mesenchymal isoform for numerous transcripts (Warzecha et al., 2009a,b). Q-RT-PCR revealed that *Esrp1* and *Esrp2* were both downregulated in *Grhl2^−/−^* epithelium compared to wild-type epithelium (Fig. 4B). Similarly, *Esrp1* was downregulated 4-fold and *Esrp2* was downregulated 3-fold in *Grhl2^−/−^* PA1, according to RNAseq. This implied that the patterns of splicing might also be perturbed in *Grhl2^−/−^* PA1 epithelium. We explored this by designing Q-RT-PCR assays specific for the epithelial and mesenchymal isoforms of four transcripts subject to ESRP-mediated splicing – *Fgfr2*, *Cd44*, *Ctnnd1* and *Enah*. In all four cases, the epithelial isoform was downregulated and the mesenchymal isoform upregulated in *Grhl2^−/−^* compared to wild-type epithelium (Fig. 4C). These results indicated that *Grhl2^−/−^* epithelium displays a partial shift towards a mesenchymal pattern of splicing.

To identify the target genes by which GRHL2 maintains the epithelial phenotype, we initially analysed four published GRHL2 chromatin immunoprecipitation (ChIP) massively parallel sequencing (ChIP-seq) datasets (Aue et al., 2015; Chung et al., 2016; Gao et al., 2013; Walentin et al., 2015), and identified regions that showed peaks in multiple datasets and contained evolutionarily conserved GRHL recognition motifs (AACCGGTT) (Ting et al., 2005). Putative GRHL2 binding sites were found in the *Mir200b*/*Mir200a/Mir429* promoter, *Mir205* enhancer, *Ovol1* promoter, *Ovol2* promoter, *Esrp1* enhancer and *Esrp2* intron 2. Notably, there were no peaks in the promoter or enhancer regions of *Zeb1* and *Zeb2*, suggesting that these are not direct targets of GRHL2 in the tissues used for ChIP-seq. We designed primers to span these putative binding sites and performed ChIP on chromatin pooled from 25 E10.5 PA1 samples. We also designed primers to amplify a predicted GRHL2 binding site in the *Zeb1* promoter, previously reported but not seen in ChIP datasets (Cieply et al., 2013). In comparison with the IgG control, the anti-GRHL2 antibody enriched precipitated chromatin for the well-defined binding sites in the known targets *Arhgef19*, *Cdh1*, *Cldn4* and *Epcam* (Fig. 4D). We also observed specific binding of GRHL2 to the predicted sites in the *Esrp1*, *Esrp2*, *Mir200b*, *Mir205*, *Ovol1* and *Ovol2* loci, but not to the *Zeb1* promoter or the negative control *Myod* promoter. Furthermore, significant GRHL2 enrichment at the binding sites near *Esrp1*, *Esrp2*, *Mir205*, *Ovol1* and *Ovol2* did not extend to loci spaced 1-1.5 kb away (Fig. S3). This suggests that GRHL2 maintains the epithelial phenotype by transactivating *Ovol1*, *Ovol2*, *Mir200b*, *Mir205*, *Esrp1* and *Esrp2*, but not by directly repressing *Zeb1*. Rather, our data suggest that GRHL2 may indirectly repress *Zeb1* by transactivating miR-200 microRNAs.

### Palate closure is restored in *Grhl2^−/−^;Zeb1^−/−^* embryos

**Fig. 5.**
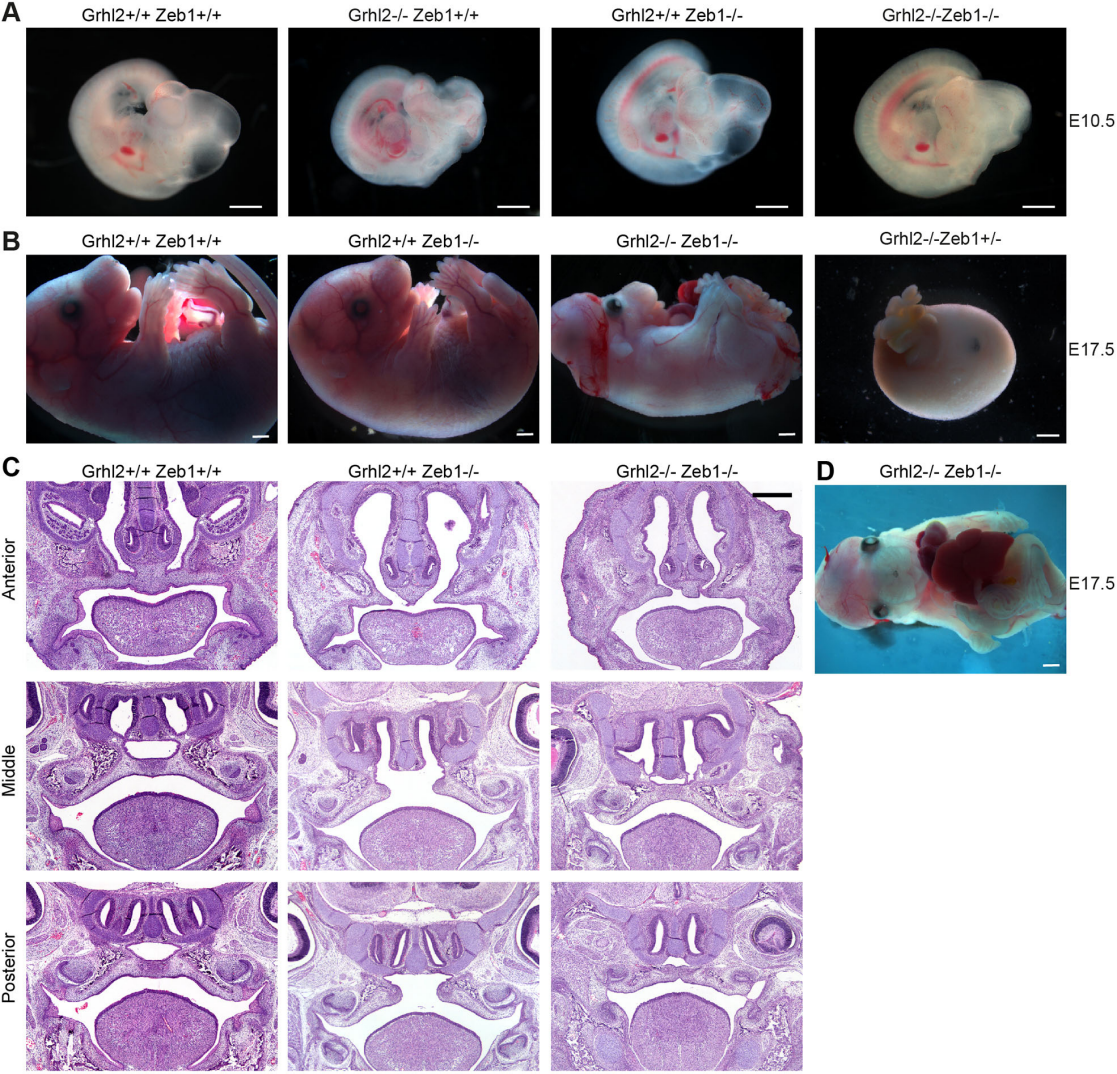
**Secondary palate closure is rescued in *Grhl2^−/−^;Zeb1^−/−^* embryos.** (A) Images at E10.5 showing cleft face in *Grhl2^−/−^* embryo and closed face in *Grhl2^−/−^;Zeb1^−/−^* embryo. (B) Images at E17.5 showing viable *Grhl2^−/−^;Zeb1^−/−^* embryo with closed face, gastroschisis, uncovered eyes, exencephaly and spina bifida, and a dead *Grhl2^−/−^;Zeb1^+/−^* embryo with a round, smooth appearance and gastroschisis. (C) Coronal sections through the palate of E17.5 embryos at anterior, middle and posterior levels. (D) Ventral view of *Grhl2^−/−^;Zeb1^−/−^* embryo at E17.5. Scale bars: 1 mm (A,B,D) and 500 μm (C).

Given the central role of *Zeb1* in the regulation of EMT, we asked whether inactivation of that gene could restore epithelial identity in the absence of GRHL2. Previous studies have shown that inactivation of *Zeb1* in mice leads to cleft palate associated with ectopic expression of E-cadherin and loss of vimentin expression in the palatal mesenchyme (Liu et al., 2008; Takagi et al., 1998). We reasoned that the balance between epithelial and mesenchymal gene expression may be restored sufficiently in *Grhl2^−/−^;Zeb1^−/−^* embryos for palate closure to occur. In order to answer this question, we intercrossed the *Grhl2^−^* and *Zeb1^−^* mouse lines. Importantly, both were on the C57BL/6J background, indicating that any phenotypic rescue that occurred would not be due to mixed genetic background. We first analysed offspring of *Grhl2^+/−^;Zeb1^+/−^*×*Grhl2^+/−^;Zeb1^+/−^* timed matings at E10.5 (Fig. 5A). Chi-squared tests revealed that all genotypes were present at expected Mendelian ratios at this stage of development (Table 1). Due to *Grhl2^−/−^;Zeb1^−/−^* embryos occurring at 1/16 in this cross, we were not able to determine whether their facial closure at E10.5 was significantly rescued. We next analysed offspring of these timed matings at E17.5 (Table 2). Chi-squared tests revealed that *Grhl2^−/−^;Zeb1^+/+^* embryos were absent by this stage of development, while all other embryos were present at expected Mendelian ratios (Table 2). Of the seven *Grhl2^−/−^;Zeb1^−/−^* embryos we collected at this stage of development, three were alive, 6 days after *Grhl2*-null mice succumb (Rifat et al., 2010). The seven *Grhl2^−/−^;Zeb1^−/−^* embryos displayed a range of phenotypes, with live embryos displaying fully penetrant exencephaly and open posterior neuropore, as seen in *Grhl2*-null embryos (Fig. 5B). They also displayed thoracogastroschisis (Fig. 5D), a phenotype observed in a previously described ENU-generated *Grhl2* mutant line that also exhibits extended survival compared to the null line (Pyrgaki et al., 2011). However, major phenotypic differences were also observed, with loss of ZEB1 allowing complete facial fusion in the four embryos that developed sufficiently for this to be determined. Twelve *Grhl2^−/−^;Zeb1^+/−^* embryos were present at E17.5, but all were dead and displaying a distinct phenotype from *Grhl2^−/−^;Zeb1^−/−^* embryos. These embryos had a ‘boiled egg’ appearance, with their only recognizable features being gastroschisis and an eye. No *Grhl2^−/−^;Zeb1^+/+^* live or dead embryos were detected at E17.5. This gene dosage effect confirmed that ZEB1 levels mediated rescue of *Grhl2^−/−^* lethality.

**Table 1.**
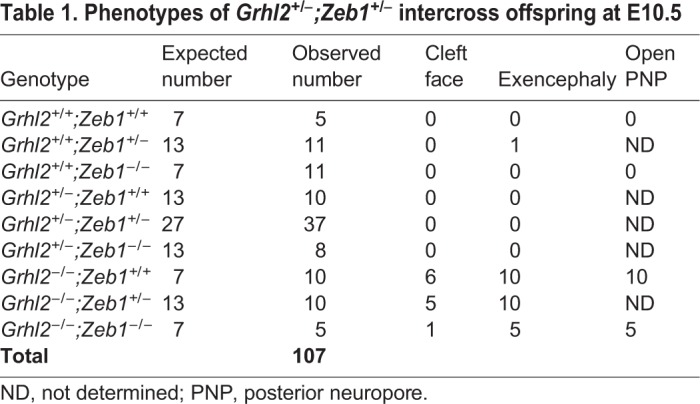
**Phenotypes of *Grhl2^+/−^;Zeb1^+/−^* intercross offspring at E10.5**

**Table 2.**
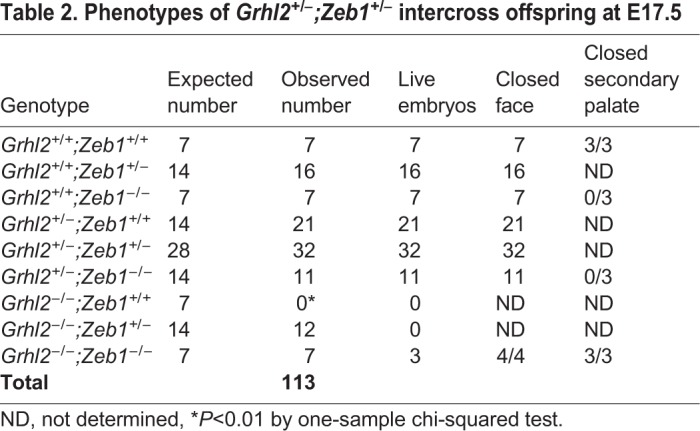
**Phenotypes of *Grhl2^+/−^;Zeb1^+/−^* intercross offspring at E17.5**

**Fig. 6.**
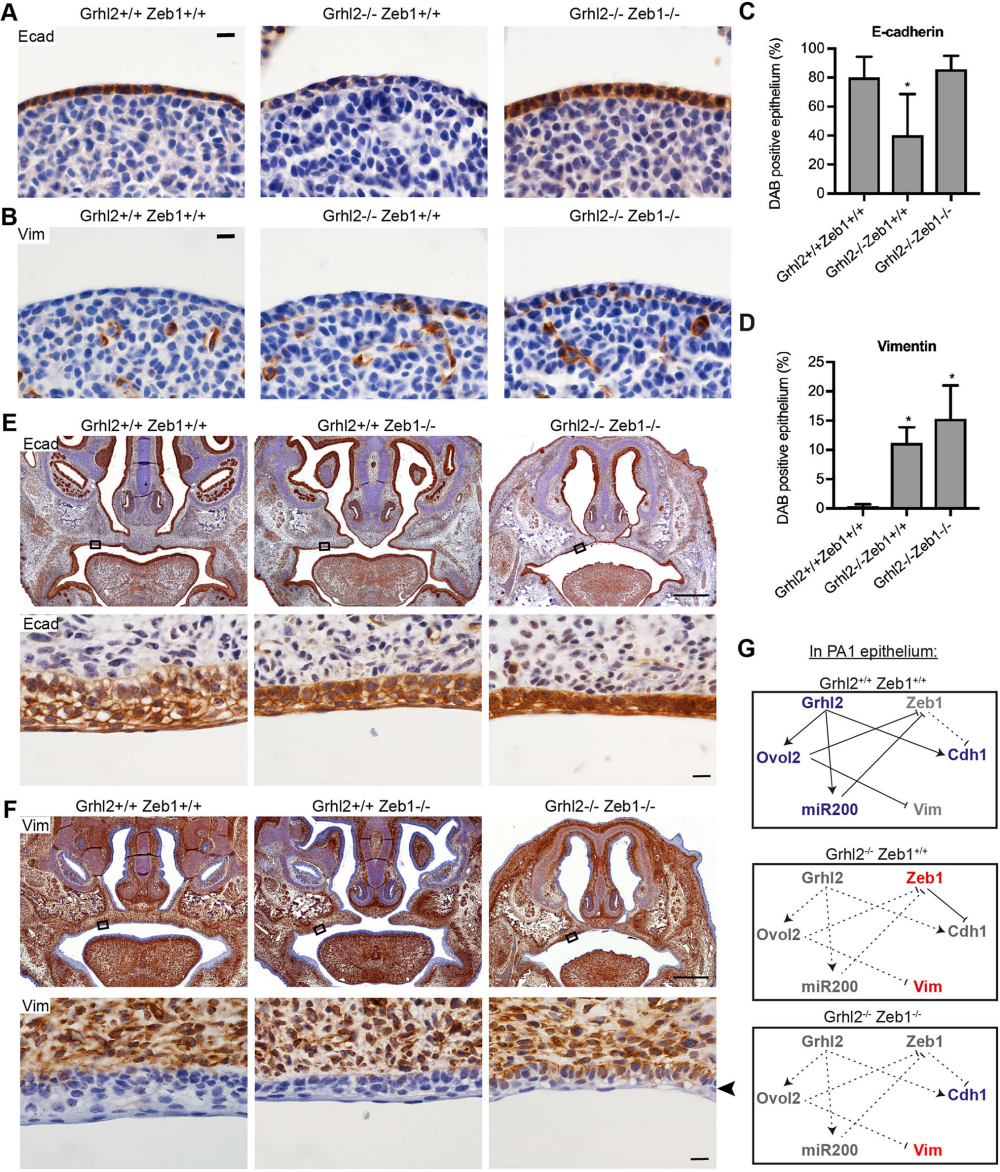
**E-cadherin expression is rescued in *Grhl2^−/−^;Zeb1^−/−^* palatal epithelium.** (A,B) Immunohistochemistry on transverse sections through E10.5 PA1 for epithelial marker E-cadherin (Ecad; A) or mesenchymal marker vimentin (Vim; B). Images are representative of four embryos. (C,D) Quantification of the proportion of E10.5 PA1 epithelium staining for Ecad (C) or Vim (D). *n*=4 embryos. Graphs show mean±s.d. *P<0.05 versus wild type by one-way ANOVA with Tukey's multiple comparison test. (E,F) Coronal sections of E17.5 embryo heads stained with immunohistochemistry for Ecad (E) or Vim (F). Bottom rows show high-magnification images of regions of the palatal epithelium indicated by boxes. Arrowhead indicates vimentin-positive *Grhl2^−/−^;Zeb1^−/−^* palatal epithelium. Images are representative of three embryos of each genotype. (G) Schematic showing the pathways by which GRHL2 maintains epithelial cellular identity in PA1 epithelium. Blue genes promote the epithelial phenotype, red genes promote the mesenchymal phenotype and grey genes are not expressed. In wild-type embryos (top), GRHL2 directly transactivates *Cdh1*, *Ovol2* and miR-200 microRNAs. The latter two factors repress *Zeb1* and *Vim*. In the absence of GRHL2 (middle), ectopically expressed ZEB1 represses *Cdh1*, and the lack of OVOL2-mediated repression leads to ectopic *Vim* expression. In the absence of both GRHL2 and ZEB1 (bottom), *Cdh1* and *Vim* are free from transcriptional repression so are both expressed. Scale bars: 500 μm (top rows in E,F) and 10 μm (A,B, bottom rows in E,F).

Live *Grhl2^−/−^;Zeb1^−/−^* embryos also displayed closure of the secondary palate, although the primary palate did not fully close (Fig. 5C; Fig. S4). The palate of *Grhl2^+/−^;Zeb1^−/−^* embryos remained fully cleft, indicating that rescue of secondary palate closure requires inactivation of both alleles of *Grhl2*. Transverse sections through E10.5 PA1 revealed that E-cadherin expression was restored to normal in *Grhl2^−/−^;Zeb1^−/−^* PA1 epithelium, although ectopic vimentin expression was still observed (Fig. 6A-D). Similarly, *Grhl2^−/−^;Zeb1^−/−^* palate epithelium co-expressed E-cadherin and vimentin at E17.5 (Fig. 6E,F). This indicated that ZEB1 likely represses the *Cdh1* locus in *Grhl2^−/−^* embryos. However, it is not clear that de-repression of this locus contributes to normalization of palate closure. Ectopic expression of E-cadherin in palatal mesenchyme was not observed in E17.5 *Zeb1^−/−^* embryos. Also, *Grhl2* mRNA was not elevated in PA1 mesenchyme of E10.5 *Zeb1^−/−^* embryos (Fig. S5). These results indicate that GRHL2 activity underlies failed closure of *Zeb1^−/−^* secondary palate.

## DISCUSSION

We have shown that the epithelial abnormalities observed in *Grhl2^−/−^* non-neural ectoderm at E8.5 (Ray and Niswander, 2016) also occur in PA1 epithelium at E10.5. This implies that other aspects of the *Grhl2* mutant mouse phenotype, such as thoracogastroschisis (Pyrgaki et al., 2011), are also a consequence of epithelial dysfunction. Furthermore, we have demonstrated that numerous key regulators of EMT are perturbed in *Grhl2^−/−^* embryos, and that the miR-200 family microRNAs, *Ovol1/Ovol2* transcription repressors and *Esrp1/Esrp2* splicing regulators are direct targets of GRHL2 in PA1 epithelium. *Zeb1/Zeb2* are de-repressed in *Grhl2^−/−^* PA1 to equivalent expression levels as in mesenchyme, while *Snai1*, *Twist1* and *Prrx1* are expressed at submesenchymal levels. Therefore, we hypothesize that upregulation of *Zeb1/Zeb2* is a driving factor in the shift from epithelial to mesenchymal phenotype in the absence of GRHL2. Our ChIP analysis of E10.5 PA1 shows that GRHL2 does not bind a previously identified binding site in the *Zeb1* promoter (Cieply et al., 2012). Rather, *Zeb1* is likely suppressed in PA1 epithelium by OVOL1/OVOL2 at the transcriptional level and by miR-200 microRNAs at the post-transcriptional level. Therefore, we hypothesize that failure to transactivate the *Ovol1*, *Ovol2* and *Mir200b/Mir200a/Mir429* loci are the key events leading to EMT in *Grhl2^−/−^* epithelium. This is consistent with the observation that GRHL2 functions as both an activator and a repressor of transcription (Aue et al., 2015). *Esrp1*, *Sostdc1*, *Fermt1*, *Tmprss2* and *Lamc2* have been previously identified as key GRHL2 target genes that suppress EMT in non-neural ectoderm (Ray and Niswander, 2016). Although *Snai1* and *Zeb2* were not detected in *Grhl2^−/−^* non-neural ectoderm (Ray and Niswander, 2016), it would be interesting to determine if *Zeb1*, *Twist1*, *Prrx1*, *Ovol1*, *Ovol2*, *Esrp2* and miR-200 microRNAs are expressed in these cells. The similarity in cellular phenotype between *Grhl2^−/−^* non-neural ectoderm at E8.5 and PA1 epithelium at E10.5 suggests that the same EMT pathways are likely perturbed in the two developmental contexts.

GRHL2 suppresses EMT and has an expression pattern inversely correlated with that of *ZEB1* in numerous cancers (Chen et al., 2016; Chung et al., 2016; Nishino et al., 2017; Paltoglou et al., 2017; Pan et al., 2017; Quan et al., 2014; Werner et al., 2013; Xiang et al., 2017). The miR-200 family of microRNAs, including *MIR141*, *MIR200A*, *MIR200B*, *MIR200C* and *MIR429*, along with *MIR205*, bind to the *ZEB1* and *ZEB2* mRNAs and inhibit their translation (Gregory et al., 2008). GRHL2 upregulates expression of the *MIR141*, *MIR200A*, *MIR200B*, *MIR200C* and *MIR429* microRNAs by binding to their regulatory elements in a number of cancers (Chen et al., 2016; Chung et al., 2016; Cieply et al., 2012). Furthermore, ZEB1 directly represses the *MIR200C/MIR141* locus in colorectal cancer cells (Burk et al., 2008). Although GRHL2 directly represses the *ZEB1* promoter in breast cancer (Cieply et al., 2012), our findings indicate that GRHL2 does not bind the *Zeb1* promoter in E10.5 PA1 epithelium. Similarly, ChIP-seq experiments showed no binding of GRHL2 to the *Zeb1* or *Zeb2* promoters in kidney, placenta, ovarian cancer or lung epithelial cells (Aue et al., 2015; Chung et al., 2016; Gao et al., 2013; Walentin et al., 2015). Interestingly, ZEB1 binds and represses the *GRHL2* promoter in breast cancer (Cieply et al., 2013; Werner et al., 2013). These observations indicate that the GRHL2/miR-200 and ZEB1/ZEB2 factors antagonize one another's expression.

Perturbation of other transcription factors in *Grhl2^−/−^* PA1 epithelium likely contributes to the mesenchymal phenotype. Like *Grhl2^−/−^* mouse embryos, *Ovol2^−/−^* embryos die mid-gestation with an open cranial neural tube (Mackay et al., 2006; Rifat et al., 2010). The phenotypic similarity between these knockout embryos suggests that *Ovol2* may be a key GRHL2 target during embryogenesis. *Ovol1^−/−^* mice present with subtle epidermal defects including abnormal hairs, expansion of epidermal progenitors and delayed acquisition of the skin barrier (Dai et al., 1998; Nair et al., 2006; Teng et al., 2007). As *Ovol2* is upregulated in *Ovol1^−/−^* epidermis, mice that lack expression of both *Ovol1* and *Ovol2* in epidermis were created (Lee et al., 2014). Keratinocytes derived from these embryos had mesenchymal characteristics including a stress fibre type of actin cytoskeleton, upregulation of ZEB1, SNAI2, vimentin, fibronectin, smooth muscle actin and N-cadherin, and downregulation of α-catenin. The epithelial phenotype of these keratinocytes was restored by knockdown of *Zeb1*. Furthermore, OVOL2 directly repressed the *Zeb1* promoter in keratinocytes. These observations support the idea that OVOL2-mediated repression of *Zeb1* is key to maintenance of the epithelial phenotype of PA1 epithelium.

The epithelial splicing regulatory proteins ESRP1 and ESRP2 have an epithelial-specific pattern of expression and are downregulated during EMT (Warzecha et al., 2009a). These factors promote splicing of pre-mRNAs into the epithelial isoform for *Fgfr2*, *Ctnnd1*, *Enah*, *Cd44* and other genes (Warzecha et al., 2009b). Interestingly, ZEB1 directly represses the ESRP1 locus in breast cancer cells (Preca et al., 2015). *Esrp1^−/−^* mice have bilateral cleft lip and palate and die neonatally, whereas *Esrp2^−/−^* mice have no overt phenotype (Bebee et al., 2015). *Esrp1^−/−^;Esrp2^−/−^* mice also display agenesis of lungs and salivary glands, but survive until E18.5. This indicates that absence of ESRP1/ESRP2 does not reconstitute all aspects of the *Grhl2^−/−^* embryo phenotype.

The secondary palate closed in viable *Grhl2^−/−^;Zeb1^−/−^* embryos but remained cleft in *Zeb1^−/−^* littermates at E17.5. This implies that GRHL2 activity in *Zeb1^−/−^* embryos disrupts closure. It is not possible to deduce the precise mechanism underlying the rescue from our work. However, the lack of ectopic *Grhl2* expression in *Zeb1^−/−^* PA1 mesenchyme at E10.5 and the absence of ectopic E-cadherin expression in *Zeb1^−/−^* palatal mesenchyme at E17.5 argue against a simple restoration of mesenchymal cell identity. Rather, the most plausible explanation is restoration of an epithelial-mesenchymal signalling pathway that promotes proliferation of palatal shelf mesenchyme. By comparison with *Grhl2^NiswNisw^* embryos that display cleft upper jaw at E18.5 (Pyrgaki et al., 2011), *Grhl2^−/−^;Zeb1^−/−^* embryos successfully closed their face and secondary palate at E17.5. This implies that ectopic expression of ZEB1 underlies failure of *Grhl2^Nisw/Nisw^* secondary palate to close. More broadly, our results imply that both epithelial and mesenchymal transcription factors are required for palate closure and that they cooperatively effect morphogenesis.

Ectopic expression of ZEB1 has been hypothesized to underlie the neural tube defects in *Grhl2* mutant mouse embryos (Ray and Niswander, 2016). We have demonstrated that this is not the case in the hindbrain and tail regions, as *Grhl2^−/−^;Zeb1^−/−^* embryos displayed fully penetrant exencephaly and open posterior neuropore at E10.5. *Grhl2^−/−^;Zeb1^−/−^* embryos sometimes survive until E17.5, while *Grhl2^−/−^* embryos die at E11.5. This indicates that ZEB1 expression in *Grhl2^−/−^* embryos underlies mid-gestation lethality. Although the cause of this early lethality is unknown, cardiac defects are a likely candidate as heart development is abnormal in *Grhl2^Nisw/Nisw^* embryos (Pyrgaki et al., 2011) and numerous fusion events occur during heart development (Ray and Niswander, 2012).

In conclusion, we have demonstrated that GRHL2 maintains epithelial cellular identity in PA1 epithelium via multiple pathways (Fig. 6G). Restoration of E-cadherin expression in *Grhl2^−/−^;Zeb1^−/−^* epithelium implies that ZEB1-mediated repression has a dominant effect on *Cdh1* expression over GRHL2-mediated transactivation. Similarly, ectopic expression of vimentin in *Grhl2^−/−^;Zeb1^−/−^* epithelium implies that OVOL2-mediated repression has a dominant effect on *Vim* expression. *Grhl2^−/−^* maxillary epithelium maintains a cellular phenotype and gene expression patterns intermediate between that of wild-type epithelium and mesenchyme. This implies that either a co-expressed transcription factor, or epigenetic marks laid down at an earlier stage of development, maintain some epithelial gene expression in these cells. It would be of interest to identify this factor, which reveals that GRHL2 is not the sole driver of epithelial phenotype during palate closure.

## MATERIALS AND METHODS

### Mouse lines

Use of animals conformed to the Australian code for the care and use of animals for scientific purposes. Experiments involving animals were approved by the Alfred Research Alliance Animal Ethics Committee (application number E/1200/2012/M). Mice carrying the *Grhl2*-null allele were maintained by heterozygote intercrosses and genotyped as described (Rifat et al., 2010). To collect embryos, mice were mated in the afternoon and females checked for a vaginal plug the following morning. Embryos were harvested at 12:00 and yolk sac DNA used for genotyping. Mice carrying a conditional allele of *Zeb1* (Brabletz et al., 2017) were crossed with mice carrying a *CMV-Cre* transgene (Schwenk et al., 1995). This deletes exon 6 of *Zeb1*, leading to a premature termination of translation and resulting in a phenotype equivalent to that of other *Zeb1^−/−^* mice (Brabletz et al., 2017). Mice carrying this *Zeb1^Δ^* allele, referred to as *Zeb1^−^* in this paper, were maintained by heterozygote intercrosses and genotyped as described (Brabletz et al., 2017). *Grhl2^−^* and *Zeb1^−^* mouse lines were both back-crossed ten times to C57BL/6J before this study commenced. *Zeb1^+/−^* mice were mated with *Grhl2^+/−^* mice and the resulting *Grhl2^+/−^;Zeb1^+/−^* offspring intercrossed as time matings. Sex of embryos was not determined. Mice carrying the *Wnt1Cre* transgene have previously been described (Danielian et al., 1998). Mice carrying the *Grhl2^fl^* allele were maintained as a homozygous line and genotyped as described (Kersbergen et al., 2018).

### Immunohistochemistry

E10.5 embryos were fixed in 4% paraformaldehyde (PFA) at 4°C overnight and then orientated in 2% low-melting-point agarose in PBS. E17.5 embryo heads were fixed similarly then processed without agarose orientation. Samples were processed using a Leica ASP300S and embedded into paraffin. Embryo heads were sectioned in a coronal orientation, while E10.5 embryos were sectioned in a transverse or coronal orientation. Five-micrometre sections were cut onto Superfrost plus slides, and immunohistochemistry was performed using standard 3,3′-diaminobenzidine (DAB) protocols. The antibodies used were anti-E-cadherin (3195, batch 02/2017, Cell Signaling Technology; 1:100), anti-vimentin (5741S, batch 04/2017, Cell Signaling Technology; 1:100) and anti-Epcam (ab71916, batch GR231753-3, Abcam; 1:1200). Validation profiles for these antibodies are available on the supplier websites. H&E staining was performed using standard protocols. Immunohistochemical staining was quantified with ImageJ software using image deconvolution and a mask for DAB-positive areas.

### Immunofluorescence

E10.5 embryos were fixed in 4% PFA, cryoprotected in 30% sucrose and embedded in OCT. Then, 10 μm sections were permeabilized with 0.25% Triton X-100 in PBS and blocked with 20% normal goat serum, 0.3% Triton X-100 in PBS. Sections were stained with rabbit monoclonal antibody against SOX9 (ab185966, Abcam) at 1:500 in 2% bovine serum albumin, 0.3% Triton X-100 in PBS overnight. After washing, sections were stained with goat polyclonal antibody against rabbit IgG conjugated to Alexa Fluor 594 (ab150080, Abcam; 1:1000) for 2 h. Sections were counterstained with 1 μg/ml Hoechst 33342 for 15 min, mounted in Vectastain mounting medium for fluorescence (H-1000, Vector Laboratories), coverslipped and cured at 4°C overnight. Sections were imaged with a Nikon A1r confocal microscope using a 20× multi-immersion objective and SOX9-positive cells were quantified using ImageJ software.

### Phalloidin staining

Embryos stored in 4% PFA were washed in PBS 3×5 min, permeabilized in 0.5% Triton X in PBS for 15 min, washed in PBS 2×2 min and blocked in 10% normal goat serum, 0.1% Triton X in PBS for 1 h. Samples were incubated in block containing rhodamine-phalloidin (1:1000; Invitrogen R415) and 1 μM Hoechst 33342 (Sigma-Aldrich) at 4°C overnight then washed with 0.1% Triton X in PBS 3×10 min. Samples were orientated onto 0.17±0.01 mm thick coverslips in 1% low-melting-point agarose in PBS and imaged using a Nikon A1r confocal microscope (Tokyo, Japan). Samples were imaged with a 60× 1.27 WI Plan Apo water immersion objective with 2× zoom. Five slices spaced 0.5 μm apart were collected using a channel series, with the depth set to image through the epithelial layer. Cell cross-sectional area and diameter of bicellular and tricellular junctions were measured using ImageJ software.

### Electron microscopy

For scanning electron microscopy (SEM), embryos were fixed overnight in 2.5% glutaraldehyde in PBS at 4°C, then rinsed in PBS for 2×10 min. Embryos were treated with 1-2.5% osmium tetroxide for 1 h then washed 2× in 0.1 M sodium cacodylate, followed by dehydration in the following washes: 50% ethanol (30 min), 70% ethanol (30 min), 95% ethanol (30 min) and 100% ethanol (2×30 min). Embryos were incubated in 50% ethanol 50% hexamethyldisilazane (HMDS; Pro Sci Tech) for 15 min, followed by 2×15 min washes in 100% HMDS. Excess HMDS was removed, and the embryos were left to dehydrate in a fume hood overnight. Embryos were mounted onto stubs with carbon tabs, sputter coated with gold using a Bal-Tec SCD 005 sputter coater and visualized using a Hitachi S-570 scanning electron microscope at 10 kV.

For transmission electron microscopy (TEM), the hind limbs and tail were removed and embryos fixed for 2 h at room temperature in 2% paraformaldehyde, 2.5% glutaraldehyde, 0.1 M sodium cacodylate, 250 μM CaCl_2_, 500 μM MgCl_2_. Embryos were then rinsed in 0.1 M sodium cacodylate and placed in 1% OsO_4_, 1.5% K_3_Fe(III)CN_6_, 0.065 M Na-cacodylate buffer (pH 7.4) for 1 h. Samples were rinsed in deionized water then dehydrated through a graded ethanol and propylene oxide series into epon resin. Samples were polymerized for 48 h at 60°C and 80 nm transverse sections through the MDP were mounted onto metal grids. Sections were imaged using a Hitachi H7500 microscope with a Gatan Multiscan 791 CCD camera between 5000× and 120,000× magnification.

### RNAseq

The PA1s of E10.5 mouse embryos were snap frozen on liquid nitrogen. Yolk sac DNA was used to determine embryo sex as described (Lambert et al., 2000). Only male samples were used for RNAseq to minimize gene expression differences due to sex. RNA from five wild-type and five *Grhl2^−/−^* PA1s was isolated using a QIAGEN micro RNeasy kit with on column DNase digestion. RNA integrity was determined using a Qubit and bioanalyser (Agilent). Libraries were constructed using the Illumina TruSeq Stranded Total RNA Library preparation (RiboZero) protocol. Sequencing was run over three HiSeq lanes using 50 bp single-end reads on HiSeq1500. Reads were aligned to the GRCm38 mouse reference genome using STAR aligner (Dobin et al., 2013), and reads were counted using featureCounts (Liao et al., 2014). Differential gene expression analysis was performed using limma-voom through the Degust interface (David Powell, Monash University, Melbourne, Australia) and trimmed mean of M-values (TMM) normalization was performed (Robinson and Oshlack, 2010). Degust software can be downloaded from http://degust.erc.monash.edu. FDR values were assigned to each gene and a cut-off of FDR<0.01 was used to select differentially expressed genes. Gene ontology analysis was performed using the Panther Overrepresentation Test and the *Mus musculus* reference list 2019-07-03 release (Ashburner et al., 2000; Mi et al., 2017) on two lists of genes: the 163 genes downregulated and the 117 genes upregulated in *Grhl2^−/−^* PA1. Bonferroni correction for multiple testing was applied. Gene set enrichment analysis was performed using GSEA v3.0 software and the molecular signatures database v5.2 (Subramanian et al., 2005).

### Q-RT-PCR

PA1 epithelium and mesenchyme were isolated from E10.5 embryos as described (Li and Williams, 2013). RNA was isolated with Trisure (Bioline) according to the manufacturer's instructions, except that precipitation was performed at −30°C overnight. RNA was reverse transcribed with the transcriptor first strand cDNA synthesis kit (Roche) for mRNA or the Quantimir RT kit (#RA420A-1, Systems Biosciences) for microRNA. Quantitative PCR (qPCR) was performed with GoTaq qPCR master mix (Promega) as duplicate 20 μl reactions or triplicate 10 μl reactions. Relative expression values were calculated using the ΔΔCT method with the normalization controls *Actb* for mRNA or *Rnu6* for microRNA. Isoform-specific Q-RT-PCR primers for *Fgfr2*, *Cd44*, *Ctnnd1* and *Enah* spanned an exon-exon junction exclusive to either the epithelial or mesenchymal isoform. Oligonucleotide primer sequences are listed in Table S5.

### ChIP

ChIP was performed essentially as described (Voss et al., 2012) on chromatin pooled from 25 E10.5 PA1s. Cross-linked chromatin was incubated with 5 μg Grhl2 antibody (HPA004820, batch B96161, Sigma-Aldrich) or 5 μg normal rabbit IgG (2729, batch 09/2009, Cell Signaling Technology). Dynabeads protein A (#10001D, Invitrogen) were used to precipitate the antibody-bound chromatin. Samples were eluted in 60 μl volume and assayed in quadruplicate Q-RT-PCR reactions using the primers listed in Table S5. The percentage input was determined by the ΔΔCt method.

### Skeletal preparations

Mouse embryo heads were fixed in 80% ethanol for 1 day, dehydrated in 96% ethanol for 1 day and incubated in 75 mM Alcian Blue, 16.5 M ethanol, 3.2 M acetic acid for 3 days. Samples were rehydrated in 70% ethanol, 40% ethanol and 15% ethanol for 2 h each then in water for 1 day. Samples were cleared in 1% KOH for 2 days, stained in 15 mM Alizarin Red, 1% KOH for 4 h then washed for 2 h three times in 1% KOH. Samples were immersed in 20% glycerol 1% KOH, 50% glycerol 1% KOH then 80% glycerol 1% KOH for 1 day each prior to imaging.

### Statistics

A sample size of four to six embryos was deemed sufficient to detect differences between genotypes based on previous experience using inbred mouse strains. No animals were excluded from the analyses. Randomization of animals to experimental groups was not performed and littermate controls were used wherever possible. Investigators were blinded to genotype when imaging embryos and histological sections. Statistical analyses were performed using Prism 7 for Mac OS X software. When comparing epidermal cell size, the Mann–Whitney *t*-test was used, as this non-parametric test does not assume a Gaussian distribution of the data. For Q-RT-PCR data, two genotypes were compared using Student's *t*-tests, not assuming consistent standard deviation, with *P*-values adjusted for multiple testing with the Holm-Sidak method. For ChIP-qPCR in Fig. 4D, two-way ANOVA was used to compare 11 different loci, with *P*-values adjusted for multiple comparisons using Sidak's method. In this case, a statistical significance cut-off of *P*<0.01 was deemed appropriate due to the slightly higher Grhl2 than IgG signal at the *Myod* locus. For ChIP-qPCR in Fig. S3, Grhl2 was compared to IgG using an unpaired Student's *t*-test. For comparison of immunohistochemical staining between three different genotypes, ordinary one-way ANOVA with Tukey's multiple comparison test was used. Chi-squared values were calculated as (observed-expected)^2^/expected and *P*-values determined for 1 degree of freedom.

## Supplementary Material

Supplementary information
