# Supplementary material for: Inactivation of Zeb1 in GRHL2-deficient mouse embryos rescues mid-gestation viability and secondary palate closure
Source: Dis Model Mech. 2020 Mar 25;13(3):dmm042218. doi: 10.1242/dmm.042218 (PMC7104862; doi:10.1242/dmm.042218)
Supplement: Supplementary information [file dmm-13-042218-s1.pdf]

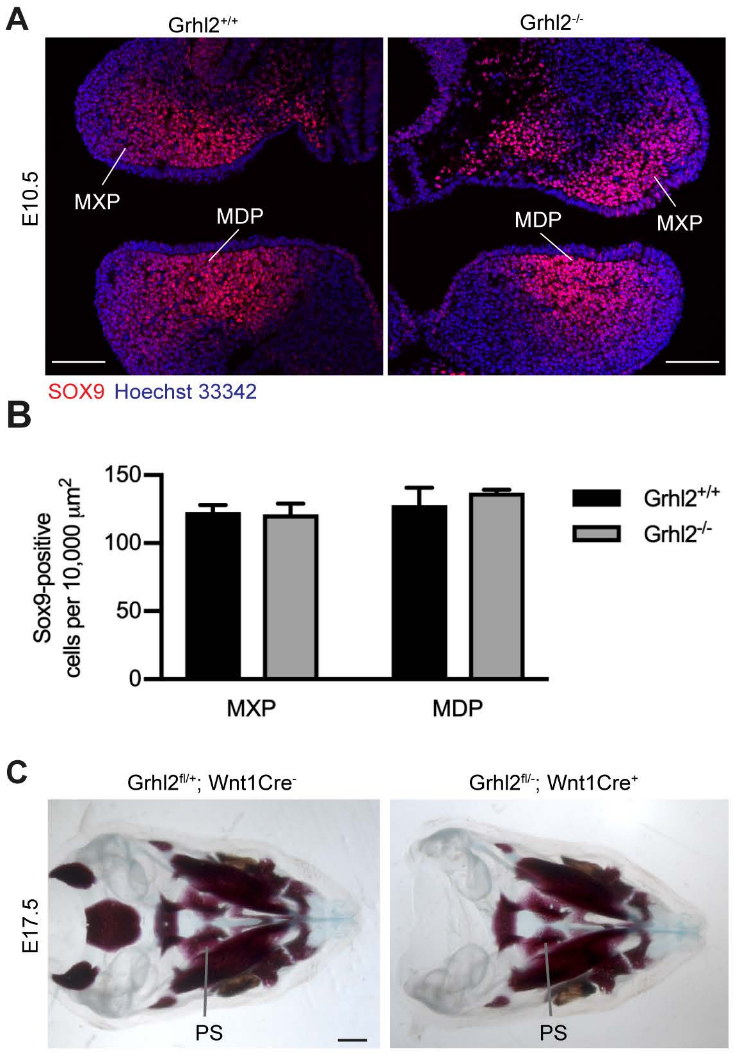

Figure S1: Grhl2 is not required in neural crest for palate closure.

A Coronal sections through E10.5 PA1 stained for SOX9 in red and DNA in blue. SOX9-positive neural crest cells are present in similar numbers in mesenchyme of wild-type and *Grhl2*$^{-/-}$ PA1. Scale bars = 100 µm. **B** Quantification of the number of SOX9-positive cells per 10,000 um^2 in the MXP or MDP mesenchyme. N=3-4 embryos. Bars show means and error bars show s.d. There was no significant difference between genotypes using an unpaired T-test. **C** Images of the ventral side of E17.5 embryo head skeletal preparations stained with alizarin red and alcian blue. Image representative of 2 *Grhl2*$^{fl/+}$*;Wnt1Cre*$^{-}$ and 3 *Grhl2*$^{fl/-}$*;Wnt1Cre*$^{+}$ embryos. Scale bar = 1mm. MXP maxillary process, MDP mandibular process, PS palatal shelf.

Disease Models & Mechanisms • Supplementary information

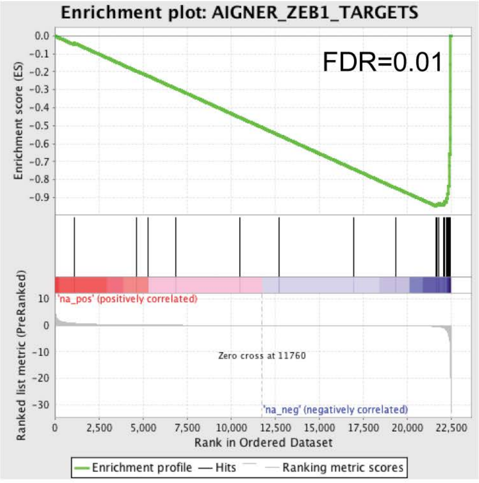

Figure S2: Genes repressed by ZEB1 are down-regulated in *Grhl2*$^{-/-}$ PA1.

Gene set enrichment analysis plot showing down-regulation of ZEB1 target genes in *Grhl2*$^{-/-}$ PA1. False discovery rate (FDR) = 0.01.

Disease Models & Mechanisms • Supplementary information

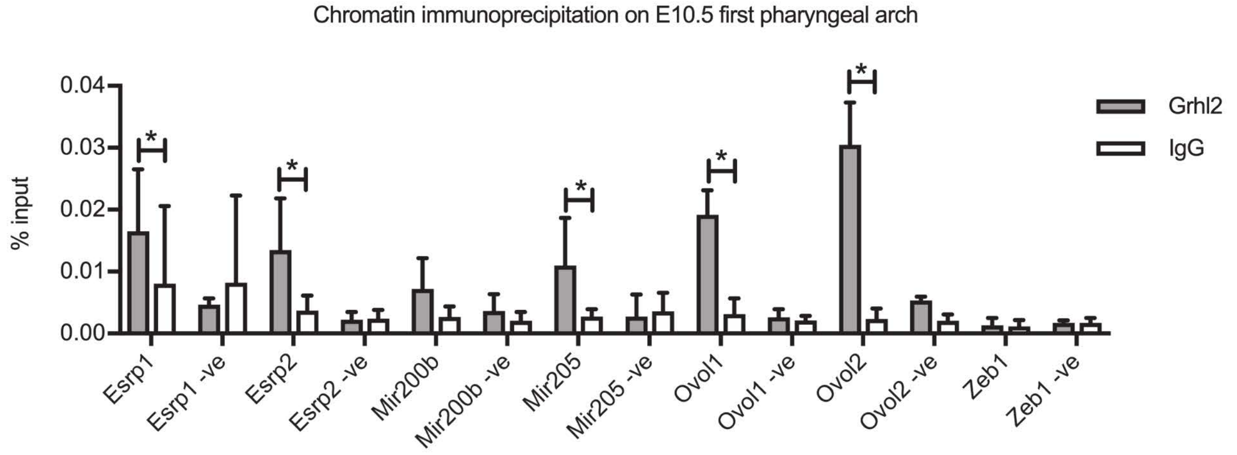

Figure S3: GRHL2 binding in E10.5 ChIP is specific.

ChIP on E10.5 PA1 with Grhl2 or IgG antibody followed by QPCR with primers spanning predicted GRHL2 binding sites or negative control (-ve) sites spaced 1-1.5 kb away. Results are representative of three independent experiments. *$p<0.05$ by unpaired t-test. Graph shows mean ±s.d. of quadruplicate QPCRs.

Disease Models & Mechanisms • Supplementary information

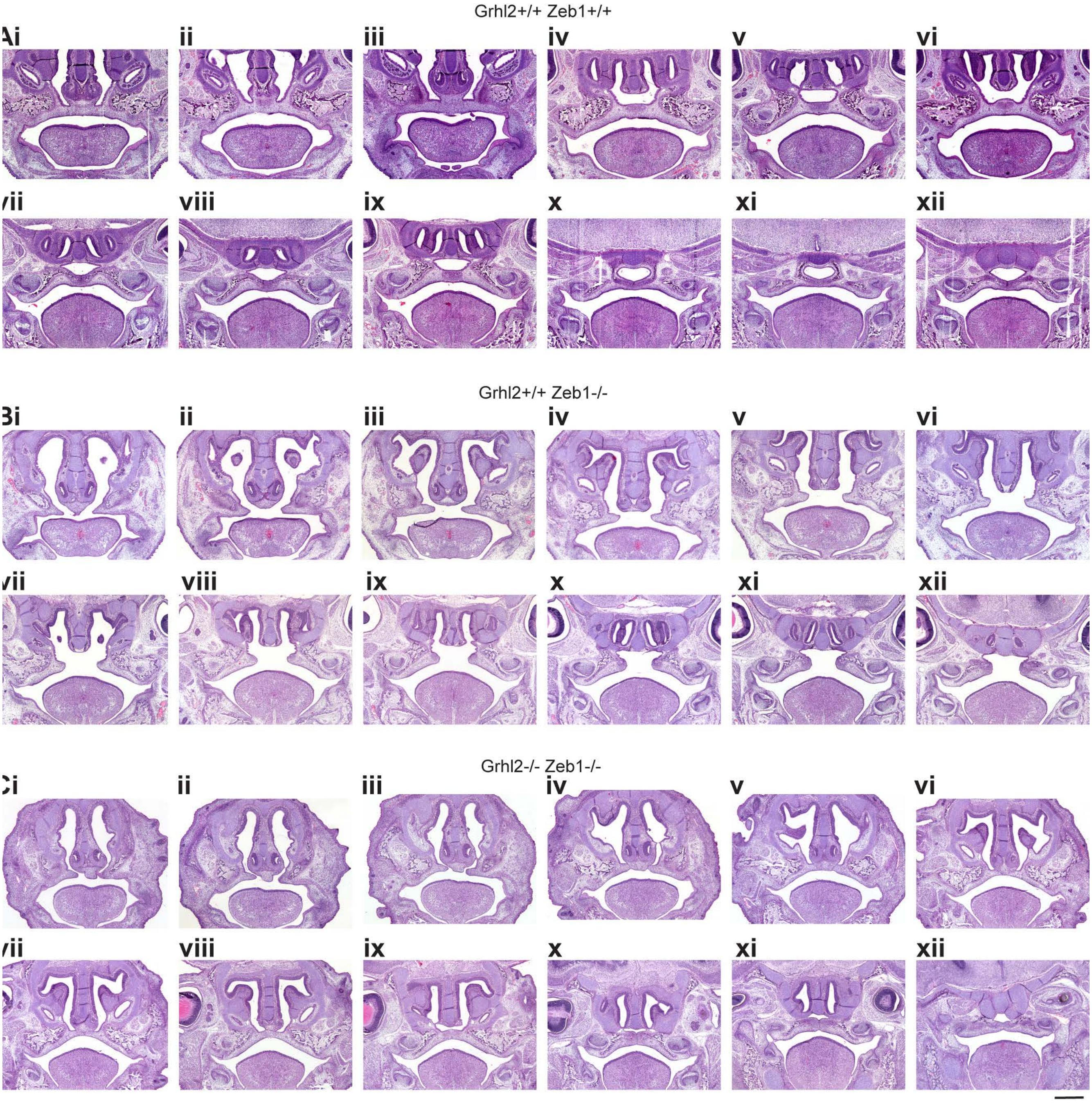

Figure S4: Serial sections show rescued palate closure in *Grhl2*$^{-/-}$*;Zeb1*$^{-/-}$ embryos. Serial coronal sections through E17.5 embryo heads showing anterior to posterior palate. **A** *Grhl2*$^{+/+}$*;Zeb1*$^{+/+}$ **B** *Grhl2*$^{+/+}$*;Zeb1*$^{-/-}$ **C** *Grhl2*$^{-/-}$*;Zeb1*$^{-/-}$. Sections **i-xii** are spaced at 100 µm intervals. Images are representative of three embryos of each genotype. Scale bar = 500 µm.

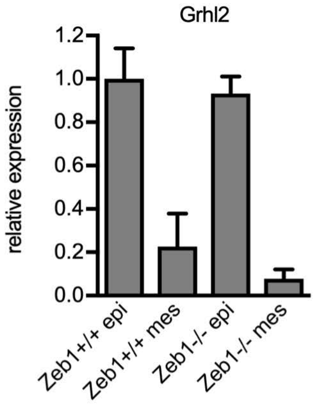

Figure S5: *Grhl2* expression is not elevated in *Zeb1*$^{-/-}$ PA1 mesenchyme.

Q-RT-PCR for *Grhl2* was performed on E10.5 PA1 epithelium and mesenchyme. N= 4 embryos of each genotype. Bars show mean and error bars show s.d.

Disease Models & Mechanisms • Supplementary information

Table S1: Genes down-regulated in *Grhl2*$^{-/-}$ PA1

Ensembl ID	Gene Name	Fold Change (Grhl2KO/WT)	False discovery rate (FDR)
ENSMUSG00000037129	Tmprss13	0.01042875	0.00001284
ENSMUSG00000028786	Tmem54	0.01225789	0.00002963
ENSMUSG00000027376	Prom2	0.01347767	0.00000523
ENSMUSG00000056973	Ces1d	0.01811811	0.00016228
ENSMUSG00000045027	Prss22	0.01956068	0.00008712
ENSMUSG00000041886	Macc1	0.02383779	0.00012180
ENSMUSG00000006143	Upk3bl	0.02452580	0.00018641
ENSMUSG00000038980	Rbbp8nl	0.02533125	0.00100901
ENSMUSG00000043088	Il17re	0.04199613	0.00016382
ENSMUSG00000022595	Lypd2	0.04347521	0.00019495
ENSMUSG00000032013	Trim29	0.05854807	0.00000210
ENSMUSG00000029055	Plch2	0.06142745	0.00000210
ENSMUSG00000037279	Ovol2	0.06165359	0.00101306
ENSMUSG00000003309	Ap1m2	0.06690453	0.00000475
ENSMUSG00000024922	Ovol1	0.07381835	0.00170953
ENSMUSG00000008601	Rab25	0.07408947	0.00014426
ENSMUSG00000028919	Arhgef19	0.07416075	0.00000624
ENSMUSG00000050520	Cldn8	0.07895543	0.00000644
ENSMUSG00000020159	Gabrp	0.08718239	0.00402726
ENSMUSG00000032292	Nr2e3	0.09052576	0.00378369
ENSMUSG00000055333	Fat2	0.09354581	0.00000097
ENSMUSG00000027186	Elf5	0.09650707	0.00883396
ENSMUSG00000057454	Lypd3	0.09786669	0.00816630
ENSMUSG00000047501	Cldn4	0.09973258	0.00000210
ENSMUSG00000031179	3830417A13Rik	0.10447151	0.00402726
ENSMUSG00000006411	Pvrl4	0.11454184	0.00114956
ENSMUSG00000028536	2610528J11Rik	0.11794925	0.00230817
ENSMUSG00000022286	Grhl2	0.12264177	0.00000210
ENSMUSG00000010080	Epn3	0.12925474	0.00000644
ENSMUSG00000031995	St14	0.13332928	0.00001578
ENSMUSG00000051397	Tacstd2	0.13461888	0.00003766
ENSMUSG00000028392	Bspry	0.13515180	0.00114956
ENSMUSG00000024479	Mal2	0.13552181	0.00292285
ENSMUSG00000039457	Ppl	0.14194177	0.00001284
ENSMUSG00000000216	Scnn1g	0.14400022	0.00204157
ENSMUSG00000022900	Ildr1	0.14507584	0.00086641
ENSMUSG00000053747	Sox14	0.14521632	0.00204157
ENSMUSG00000053886	Sh2d4a	0.15596843	0.00704642
ENSMUSG00000061527	Krt5	0.15953955	0.00646324
ENSMUSG00000030800	Prss8	0.16897038	0.00008712
ENSMUSG00000028115	Bnipl	0.17008616	0.00024676
ENSMUSG00000074277	Phldb3	0.17059179	0.00076754
ENSMUSG00000045394	Epcam	0.17517541	0.00000798
ENSMUSG00000000303	Cdh1	0.18034420	0.00001284

ENSMUSG00000034382	AI661453	0.20318844	0.00001898
ENSMUSG00000034308	Sdr42e1	0.20912629	0.00119690
ENSMUSG00000027356	Fermt1	0.21126118	0.00000121
ENSMUSG00000037600	Kdf1	0.22898444	0.00019828
ENSMUSG00000054065	Pkp3	0.23004457	0.00001200
ENSMUSG00000036687	Tmem184a	0.23346210	0.00391623
ENSMUSG00000041782	Lad1	0.24008499	0.00018968
ENSMUSG00000022382	Wnt7b	0.24434985	0.00119235
ENSMUSG00000026639	Lamb3	0.24998459	0.00490459
ENSMUSG00000023906	Cldn6	0.25378052	0.00008879
ENSMUSG00000044279	Crb3	0.26071914	0.00347080
ENSMUSG00000039529	Atp8b1	0.26276223	0.00012180
ENSMUSG00000050854	Tmem125	0.26545755	0.00146051
ENSMUSG00000013611	Snx31	0.26885841	0.00209095
ENSMUSG00000040728	Esrp1	0.27298978	0.00016382
ENSMUSG00000028841	Cnksr1	0.27634817	0.00112757
ENSMUSG00000074923	Pak6	0.28490873	0.00127602
ENSMUSG00000055976	Cldn23	0.28976713	0.00569818
ENSMUSG00000030873	Scnn1b	0.29398962	0.00843697
ENSMUSG00000001583	Tnk1	0.29885471	0.00347080
ENSMUSG00000026994	Galnt3	0.30008594	0.00029446
ENSMUSG00000020838	Slc6a4	0.30081329	0.00231539
ENSMUSG00000032358	Fam83b	0.30521684	0.00071769
ENSMUSG00000084128	Esrp2	0.30872825	0.00000624
ENSMUSG00000028865	Cd164l2	0.31300538	0.00925019
ENSMUSG00000018569	Cldn7	0.32148786	0.00028219
ENSMUSG00000026668	Ucma	0.32174580	0.00816630
ENSMUSG00000026971	Itgb6	0.32301381	0.00126446
ENSMUSG00000001672	Marveld3	0.32407576	0.00905862
ENSMUSG00000023039	Krt7	0.32495298	0.00404390
ENSMUSG00000018581	Dnah11	0.32779336	0.00381960
ENSMUSG00000022408	Fam83f	0.33010328	0.00051453
ENSMUSG00000057615	Ldoc1	0.33420026	0.00316936
ENSMUSG00000021604	Irx4	0.33515957	0.00056488
ENSMUSG00000034282	Evpl	0.34113269	0.00028219
ENSMUSG00000032092	Mpzl2	0.34530189	0.00021393
ENSMUSG00000019866	Aim1	0.34765154	0.00005739
ENSMUSG00000021340	Gpld1	0.35400347	0.00483717
ENSMUSG00000058600	Rpl30	0.37166902	0.00017099
ENSMUSG00000047281	Sfn	0.37237278	0.00042625
ENSMUSG00000030739	Myh14	0.37979701	0.00121918
ENSMUSG00000045871	Slitrk6	0.40353283	0.00974449
ENSMUSG00000017607	Tns4	0.40454179	0.00014426
ENSMUSG00000026890	Lhx6	0.40828588	0.00031988
ENSMUSG00000034584	Exph5	0.41286430	0.00016120
ENSMUSG00000029859	Epha1	0.41999892	0.00188004
ENSMUSG00000024331	Dsc2	0.42456523	0.00091730
ENSMUSG00000032776	Mctp2	0.42611202	0.00397853

ENSMUSG00000054889	Dsp	0.42815072	0.00000794
ENSMUSG00000091243	Vgll3	0.43911755	0.00076754
ENSMUSG00000020758	Itgb4	0.44764470	0.00211914
ENSMUSG00000044393	Dsg2	0.44989612	0.00076227
ENSMUSG00000039813	Tbc1d2	0.45441554	0.00022893
ENSMUSG00000033998	Kcnk1	0.45736751	0.00018641
ENSMUSG00000030510	Cers3	0.46994748	0.00609235
ENSMUSG00000031870	Pgr	0.47170069	0.00209095
ENSMUSG00000096225	Lhx8	0.48314879	0.00001898
ENSMUSG00000035498	Cdcp1	0.48845768	0.00290260
ENSMUSG00000028640	Tfap2c	0.50144955	0.00071858
ENSMUSG00000026479	Lamc2	0.50713539	0.00230219
ENSMUSG00000029330	Cds1	0.50985861	0.00873137
ENSMUSG00000031380	Figf	0.51001175	0.00122154
ENSMUSG00000021636	Marveld2	0.51618668	0.00861956
ENSMUSG00000068876	Cgn	0.52086206	0.00190916
ENSMUSG00000027315	Spint1	0.52394092	0.00527843
ENSMUSG00000007888	Crlf1	0.54065941	0.00453778
ENSMUSG00000029149	Krtcap3	0.54798414	0.00861956
ENSMUSG00000030217	Art4	0.55173831	0.00121413
ENSMUSG00000030688	Stard10	0.55201559	0.00091316
ENSMUSG00000005251	Ripk4	0.55300184	0.00052487
ENSMUSG00000043003	Rasef	0.55570692	0.00209095
ENSMUSG00000032561	Acpp	0.56378560	0.00816630
ENSMUSG00000070305	Mpzl3	0.56838590	0.00220965
ENSMUSG00000021062	Rab15	0.56988053	0.00101441
ENSMUSG00000048747	E130114P18Rik	0.57260554	0.00756277
ENSMUSG00000024511	Rab27b	0.57298877	0.00715780
ENSMUSG00000034435	Tmem30b	0.57583037	0.00054178
ENSMUSG00000030208	Emp1	0.57917791	0.00207737
ENSMUSG00000029032	Arhgef16	0.60211624	0.00050122
ENSMUSG00000033227	Wnt6	0.60343685	0.00018968
ENSMUSG00000017057	Il13ra1	0.61186822	0.00230817
ENSMUSG00000024868	Dkk1	0.62520266	0.00527843
ENSMUSG00000026972	Arrdc1	0.62529059	0.00056373
ENSMUSG00000051279	Gdf6	0.62626958	0.00120688
ENSMUSG00000051323	Pcdh19	0.62833170	0.00756277
ENSMUSG00000048450	Msx1	0.63151089	0.00551701
ENSMUSG00000074121	Ntf5	0.65498666	0.00870000
ENSMUSG00000000093	Tbx2	0.65672912	0.00101306
ENSMUSG00000073988	Ttpa	0.65789172	0.00174336
ENSMUSG00000033542	Arhgef5	0.66143137	0.00041672
ENSMUSG00000056380	Gpr50	0.67389483	0.00101049
ENSMUSG00000035964	Tmem59l	0.67433938	0.00649329
ENSMUSG00000015133	Lrrk1	0.68039282	0.00031534
ENSMUSG00000043496	Tril	0.68129502	0.00502307
ENSMUSG00000025915	Sgk3	0.69306642	0.00402726
ENSMUSG00000032261	Sh3bgrl2	0.72050204	0.00957437

Disease Models & Mechanisms • Supplementary information

ENSMUSG00000019851	Perp	0.72128268	0.00640783
ENSMUSG00000035407	Kank4	0.72378862	0.00034617
ENSMUSG00000018604	Tbx3	0.73319259	0.00316936
ENSMUSG00000038910	Plcl2	0.74693618	0.00097135
ENSMUSG00000024981	Acsl5	0.76864705	0.00391623
ENSMUSG00000002068	Ccne1	0.77101757	0.00767626
ENSMUSG00000028654	Mycl	0.79219043	0.00105981
ENSMUSG00000032221	Mns1	0.79467561	0.00916732
ENSMUSG00000025932	Eya1	0.79650085	0.00084298
ENSMUSG00000028212	Ccne2	0.80666923	0.00579675
ENSMUSG00000019960	Dusp6	0.80691210	0.00843697
ENSMUSG00000018585	Atox1	0.80967677	0.00470561
ENSMUSG00000025068	Gsto1	0.81148474	0.00165399
ENSMUSG00000036528	Ppfibp2	0.81150341	0.00973878
ENSMUSG00000001864	Aif1l	0.81717740	0.00306096
ENSMUSG00000000753	Serpinf1	0.81797293	0.00767626
ENSMUSG00000029125	Stx18	0.82719111	0.00905862
ENSMUSG00000031479	Vps36	0.82961248	0.00431824
ENSMUSG00000005667	Mthfd2	0.83082749	0.00739323
ENSMUSG00000032252	Glce	0.83796409	0.00905862
ENSMUSG00000036781	Rps27l	0.84876987	0.00744936
ENSMUSG00000026889	Rbm18	0.86446734	0.00845819
ENSMUSG00000031701	Dnaja2	0.86773981	0.00551701

Table S2: Genes upregulated in *Grhl2*$^{-/-}$ PA1

Ensembl ID	Gene Name	Fold Change (Grhl2KO/WT)	False discovery rate (FDR)
ENSMUSG00000070870	Cryge	20.93200502	0.00935863
ENSMUSG00000025952	Crygc	11.13315014	0.00212075
ENSMUSG00000031073	Fgf15	8.20879719	0.00870000
ENSMUSG00000042115	Klhdc8a	7.99758484	0.00979781
ENSMUSG00000032085	Tagln	5.63269220	0.00016120
ENSMUSG00000058669	Nkx2-9	4.64343132	0.00861956
ENSMUSG00000025586	Cpeb1	4.57124494	0.00715780
ENSMUSG00000048096	Lmod1	4.44006082	0.00010809
ENSMUSG00000032269	Htr3a	3.85897266	0.00146051
ENSMUSG00000020734	Grin2c	3.71175988	0.00146051
ENSMUSG00000055874	Foxi3	3.28709016	0.00591308
ENSMUSG00000048562	Sp8	3.21504605	0.00095689
ENSMUSG00000022144	Gdnf	3.10256959	0.00019359
ENSMUSG00000028661	Epha8	2.60265002	0.00204782
ENSMUSG00000074607	Tox2	2.59578899	0.00460869
ENSMUSG00000001663	Gstt1	2.44241251	0.00588191
ENSMUSG00000022887	Masp1	2.39138959	0.00174336
ENSMUSG00000001930	Vwf	2.34180644	0.00670994
ENSMUSG00000035513	Ntng2	2.17579448	0.00887497
ENSMUSG00000027358	Bmp2	2.16605081	0.00072499
ENSMUSG00000034205	Loxl2	2.15180921	0.00217454
ENSMUSG00000041912	Tdrkh	2.13708255	0.00673460
ENSMUSG00000029309	Sparcl1	2.13045563	0.00744936
ENSMUSG00000006445	Epha2	2.09849930	0.00000210
ENSMUSG00000067818	Myl9	2.07678761	0.00756277
ENSMUSG00000031273	Col4a6	2.07446266	0.00027094
ENSMUSG00000028370	Pappa	2.06306098	0.00222866
ENSMUSG00000006930	Hap1	2.05157753	0.00497179
ENSMUSG00000009772	Nuak2	2.04785892	0.00282357
ENSMUSG00000031654	Cbln1	2.02634512	0.00209095
ENSMUSG00000041957	Pkp2	2.02245999	0.00650198
ENSMUSG00000036834	Plch1	2.00941550	0.00089061
ENSMUSG00000027584	Oprl1	1.96400744	0.00453778
ENSMUSG00000044220	Nkx2-3	1.93228483	0.00756277
ENSMUSG00000020427	Igfbp3	1.91211825	0.00018641
ENSMUSG00000031517	Gpm6a	1.90186644	0.00316936
ENSMUSG00000024087	Cyp1b1	1.90148547	0.00902323
ENSMUSG00000029765	Plxna4	1.88187828	0.00744936
ENSMUSG00000022489	Pde1b	1.84416914	0.00219172
ENSMUSG00000042734	Ttc9	1.81769105	0.00237283
ENSMUSG00000069170	Adgrv1	1.78601625	0.00816630
ENSMUSG00000006567	Atp7b	1.78325235	0.00861956
ENSMUSG00000027750	Postn	1.78107640	0.00630807
ENSMUSG00000041592	Sdk2	1.74620656	0.00101306

ENSMUSG00000054364	Rhob	1.74401098	0.00692532
ENSMUSG00000035095	Fam167a	1.70085997	0.00451050
ENSMUSG00000031825	Crispld2	1.67816596	0.00470562
ENSMUSG00000022367	Has2	1.65589158	0.00016854
ENSMUSG00000031075	Ano1	1.65336249	0.00178688
ENSMUSG00000069763	Tmem100	1.61941627	0.00608161
ENSMUSG00000023067	Cdkn1a	1.61836928	0.00883396
ENSMUSG00000024008	Cpne5	1.60347761	0.00292285
ENSMUSG00000058897	Col25a1	1.57936902	0.00130637
ENSMUSG00000049336	Tenm2	1.57699614	0.00453778
ENSMUSG00000039167	Adgrl4	1.57362576	0.00905862
ENSMUSG00000022206	Npr3	1.57105814	0.00377255
ENSMUSG00000024304	Cdh2	1.56733886	0.00127740
ENSMUSG00000034903	Cobll1	1.55192173	0.00861956
ENSMUSG00000074622	Mafb	1.54298282	0.00488561
ENSMUSG00000040289	Hey1	1.53517303	0.00417367
ENSMUSG00000037239	Spred3	1.53212784	0.00307079
ENSMUSG00000032452	Clstn2	1.51003095	0.00762493
ENSMUSG00000030376	Slc8a2	1.50759374	0.00732989
ENSMUSG00000043639	Rbm20	1.50121193	0.00488561
ENSMUSG00000017009	Sdc4	1.48073688	0.00497179
ENSMUSG00000032334	Loxl1	1.47882873	0.00609235
ENSMUSG00000018593	Sparc	1.47452739	0.00003151
ENSMUSG00000032850	Rnft2	1.46937662	0.00922925
ENSMUSG00000028464	Tpm2	1.46568506	0.00417367
ENSMUSG00000031626	Sorbs2	1.46364864	0.00306096
ENSMUSG00000050373	Snx21	1.45009260	0.00347080
ENSMUSG00000021009	Ptpn21	1.44553923	0.00861956
ENSMUSG00000001870	Ltbp1	1.44440455	0.00049766
ENSMUSG00000026185	Igfbp5	1.43831432	0.00862263
ENSMUSG00000047497	Adamts12	1.43823569	0.00222866
ENSMUSG00000007038	Neu1	1.41145423	0.00209095
ENSMUSG00000003534	Ddr1	1.40734217	0.00028219
ENSMUSG00000031274	Col4a5	1.40081904	0.00023310
ENSMUSG00000020032	Nuak1	1.39635236	0.00889694
ENSMUSG00000074505	Fat3	1.39578881	0.00907772
ENSMUSG00000054708	Ankrd24	1.39152317	0.00999390
ENSMUSG00000046329	Slc25a23	1.38475955	0.00071769
ENSMUSG00000033453	Adamts15	1.36682355	0.00704642
ENSMUSG00000062031	Athl1	1.36410375	0.00636037
ENSMUSG00000029287	Tgfbr3	1.36021369	0.00316936
ENSMUSG00000038267	Slc22a23	1.35278049	0.00453778
ENSMUSG00000052105	Mtcl1	1.34430890	0.00441082
ENSMUSG00000039706	Ldb2	1.34321370	0.00521437
ENSMUSG00000004891	Nes	1.31724674	0.00910610
ENSMUSG00000052713	Zfp608	1.31701863	0.00715780
ENSMUSG00000041633	Kctd12b	1.30489057	0.00469979
ENSMUSG00000052911	Lamb2	1.30219420	0.00710286

ENSMUSG00000008855	Hdac5	1.30083249	0.00281834
ENSMUSG00000040502	38411	1.28889340	0.00905862
ENSMUSG00000034993	Vat1	1.28670263	0.00770755
ENSMUSG00000031558	Slit2	1.28096181	0.00816630
ENSMUSG00000027111	Itga6	1.27514996	0.00054178
ENSMUSG00000029070	Mxra8	1.27330064	0.00553860
ENSMUSG00000027669	Gnb4	1.27178909	0.00204157
ENSMUSG00000034037	Fgd5	1.26580719	0.00733749
ENSMUSG00000006403	Adamts4	1.26455568	0.00770755
ENSMUSG00000025579	Gaa	1.26366282	0.00527535
ENSMUSG00000021806	Nid2	1.26131517	0.00114956
ENSMUSG00000061353	Cxcl12	1.25029847	0.00265696
ENSMUSG00000026478	Lamc1	1.24845867	0.00803935
ENSMUSG00000023830	Igf2r	1.24230477	0.00921781
ENSMUSG00000040841	Six5	1.24100084	0.00347080
ENSMUSG00000031555	Adam9	1.24050786	0.00174336
ENSMUSG00000013236	Ptprs	1.23393543	0.00269740
ENSMUSG00000025278	Flnb	1.22603941	0.00488561
ENSMUSG00000019467	Arhgef25	1.22574663	0.00905862
ENSMUSG00000075254	Heg1	1.22527805	0.00796836
ENSMUSG00000031367	Ap1s2	1.22508854	0.00526471
ENSMUSG00000007041	Clic1	1.21637312	0.00678991
ENSMUSG00000029869	Ephb6	1.19696741	0.00935863
ENSMUSG00000002900	Lamb1	1.17427310	0.00816630
ENSMUSG00000032666	1700025G04Rik	1.16811231	0.00765558

Table S3: Genes down-regulated in *Grhl2*$^{-/-}$ PA1 from key ontologies

Gene Ontology	Genes
desmosome organization (GO:0002934)	Pkp3, Dsg2, Dsp, Perp
bi-cellular tight junction assembly (GO:0070830)	Marveld2, Cgn, Grhl2, Marveld3, Cdh1
multicellular organismal water homeostasis (GO:0050891)	Scnn1b, Tmprss13, Cldn4, Sfn, Kdf1, Scnn1g, Cdh1
regulation of epidermal cell differentiation (GO:0045604)	Esrp1, Mycl, Ovol2, Sfn, Kdf1, Grhl2
odontogenesis of dentin-containing tooth (GO:0042475)	Wnt6, Itgb4, Wnt7b, Msx1, Lhx8, Dsp, Perp
keratinocyte differentiation (GO:0030216)	Evpl, Cers3, St14, Tfap2c, Exph5, Sfn, Kdf1, Dsp, Ppl
epithelial cell development (GO:0002064)	Marveld2, St14, Tfap2c, Exph5, Sfn, Slitrk6, Cgn, Kdf1, Rab25, Wnt7b, Grhl2, Pgr, Cdh1
morphogenesis of a branching epithelium (GO:0061138)	Esrp1, St14, Wnt6, Tfap2c, Spint1, Tbx3, Esrp2, Grhl2, Eya1, Pgr, Dsp
epithelial tube morphogenesis (GO:0060562)	Esrp1, St14, Wnt6, Tfap2c, Ovol2, Spint1, Tbx3, Esrp2, Grhl2, Tbx2, Eya1, Pgr, Dsp, Tacstd2
cell adhesion (GO:0007155)	Lypd3, Itgb6, Lamb3, Pcdh19, Mpzl2, Epha1, Pkp3, Epcam, Itgb4, Lamc2, Wnt7b, Mpzl3, Grhl2, Pvrl4, Fat2, Dsg2, Dsc2, Fermt1, Cdh1, Dsp, Perp

Table S4: Genes up-regulated in *Grhl2*$^{-/-}$ PA1 from key ontologies

Gene Ontology	Genes
cardiac epithelial to mesenchymal transition (GO:0060317)	Has2, Tmem100, Tgfbr3, Bmp2, Hey1
osteoblast differentiation (GO:0001649)	Tgfbr3, Igfbp5, Epha2, Bmp2, Igfbp3, Hdac5, Hey1
cell-substrate adhesion (GO:0031589)	Itga6, Nid2, Adam9, Ddr1, Lamb1, Adamts12, Vwf, Lamc1, Lamb2
cell-cell adhesion via plasma-membrane adhesion molecules (GO:0098742)	Clstn2, Ptprs, Tenm2, Cdh2, Ntng2, Sparcl1, Fat3, Sdk2, Cbln1
axon guidance (GO:0007411)	Slit2, Epha8, Cxcl12, Tenm2, Gdnf, Epha2, Ephb6, Lamb2, Ntng2, Plxna4
extracellular matrix organization (GO:0030198)	Has2, Crispld2, Col4a6, Postn, Cyp1b1, Col4a5, Lamb1, Loxl2, Lamc1, Col25a1, Lamb2
regulation of synapse organization (GO:0050807)	Clstn2, Ptprs, Sparc, Neu1, Sorbs2, Cdh2, Ntng2, Sparcl1, Gpm6a, Cbln1
response to growth factor (GO:0070848)	Adam9, Has2, Tmem100, Tgfbr3, Ltbp1, Adamts12, Sparc, Gdnf, Epha2, Bmp2, Fgf15, Hap1
blood vessel morphogenesis (GO:0048514)	Slit2, Heg1, Has2, Tmem100, Cxcl12, Tgfbr3, Cyp1b1, Loxl2, Rhob, Epha2, Cdh2, Hey1
enzyme linked receptor protein signaling pathway (GO:0007167)	Epha8, Adam9, Tmem100, Col4a6, Tgfbr3, Col4a5, Ddr1, Ltbp1, Gdnf, Epha2, Bmp2, Ephb6, Fgf15, Hap1

Table S5: Oligonucleotide primer sequences.

Use	Target gene	Direction	Sequence
QRTPCR	Cdh1	forward	GGACCGAGAGAGTTACCCT
		reverse	CCCTGATACGTGCTTGGGT
	Cdh2	forward	CAGCCCCTTCTCAATGTGAAAT
		reverse	CTTGAAATCTGCTGGCTCGC
	Epcam	forward	CATTTGCTCCAAACTGGCGT
		reverse	TTGTTCTGGATCGCCCCTTC
	Vim	forward	TGCACGATGAAGAGATCCAGG
		reverse	CTCCTGGAGGTTCTTGGCAG
	Grhl2	forward	TGCAACAACTCCTCTGATGG
		reverse	TAAACCTGTCCGGTCCTCTG
	Ovol1	forward	AGCCTTCGAGACTCCAGCTA
		reverse	AGGTCACCTTCATCTTGGTTCG
	Ovol2	forward	AACTCCAGAGCTTCACGACG
		reverse	TGTGCCGGTGGTAAACTTGA
	Grhl3	forward	AGCCAACCAGAGACGGATC
		reverse	AGGCCTCGTCCTCATTACTG
	Prrx1	forward	GGAGCAACCCATCGTACCTC
		reverse	CATGGCGCTGTACGGAGA
	Snai1	forward	TAGGTCGCTCTGGCCAACAT
		reverse	CTGGAAGGTGAACTCCACACA
	Twist1	forward	CCGGAGACCTAGATGTCATTGT
		reverse	CCACGCCCTGATTCTTGTGA
	Zeb1	forward	GCCAAACGGAAACCAGGATG
		reverse	GGCGTGGAGTCAGAGTCATT
	Zeb2	forward	TACCTTCAGCGAAGCGACAC
		reverse	GTTCCAGGTGGCAGGTCATT
	Actb	forward	GATATCGCTGCGCTGGTCGTC
		reverse	ACGCAGCTCATTGTAGAAGGTGTGG
	Esrp2	forward	TCTACGACCTCCGCAGAGAA
		reverse	CTAGTCCCAAGTCCTGTGCC
	Esrp1	forward	CTGGCACCGTGGTCAGAAT
		reverse	TCCTCGGTTGCATACTGGTAAC
	miR-141-3p	for	TAACACTGTCTGGTAAAGATGG
	miR-200a-3p	for	TAACACTGTCTGGTAACGATGT
	miR-200b-3p	for	TAATACTGCCTGGTAATGATGA
	miR-200c-3p	for	TAATACTGCCGGGTAATGATGGA
	miR-429-3p	for	TAATACTGTCTGGTAATGCCGT
	Fgfr2(epithelial)	forward	AGGTTTACAGCGATGCCCAG
		reverse	TTATCCCCGAGTGCTTCAGGA
	Fgfr2 (mesenchymal)	forward	CGTGCTTGGCGGGTAATTCT
		reverse	CTTCTCTCTCACAGGCGCTGG
	Cd44 (epithelial)	forward	ACATTACATGGAGAGCCGGA
		reverse	CGCCGCTCTTAGTGCTAGAT
	Cd44 (mesenchymal)	forward	CTTGGCCACCAGAGATCGAG
		reverse	TGTCCTGGTTCGCACTTGAG
	Ctnnd (epithelial)	forward	ATTTGAGCTCTCTCCTTCCTGC

		reverse	GGTCCCCTCACTTCACACTG
	Ctnnd1 (mesenchymal)	forward	CGAACCTCGCTGGATTTGTC
		reverse	AACCGGCCGTTCTCAAATGT
	Enah (epithelial)	forward	ACTGCTAAGGCCCCATCAAC
		reverse	GGTGTGGATTTGGGTCTGGA
	Enah (mesenchymal)	forward	CTCCAGACGGGATTCTCCAAG
		reverse	AGGTGTGGATTTGGGTCTGTG
CHIP	MyoD	forward	ACACGACTGCTTTCTTCACCA
		reverse	AAGCCGTGAGAGTCGTCTTA
	Arhgef19	forward	GAGCTTCTGGGAGGCATAGG
		reverse	CTGTCTCAACGTGCCTGCT
	Cdh1	forward	TGGTTGAAAGTTCCCCTAAGC
		reverse	CCGTTTGCAAAGCAGTGTT
	Cldn4	forward	CTTTGTTGGCCCAAGCTC
		reverse	CCACAGGTGCTGCAGTTAAA
	Epcam	forward	TGCTTTTTCTCCCGCCAGTAG
		reverse	CTGTTCAGCCCCCAGTTTGG
	Esrp1	forward	CGAGCAACCGAGATGGTCA
		reverse	GCGGGGAATCTGGAAAAACCA
	Esrp2	forward	AGGCCTTTTACTTCCCAGGC
		reverse	GGACAGGGAGTGGAGACTCA
	Mir200b	forward	TGCCTCGATACTGGGGGTG
		reverse	CTCTCCCTACATGGTGTTCTCA
	Mir205	forward	AACTTGGCTGAGAGGTGCAT
		reverse	GAAGCTGGAAAGAGAGGGGG
	Ovol1	forward	CGGTGACAACCCACCTATTT
		reverse	GGGTGCTTCTGGGTGTGG
	Ovol2	forward	CATTCATGCCTAAGGAAGGGCT
		reverse	GCCCTGCAACATAACGGTG
	Zeb1	forward	GCAAAACTTTTCCCTCGCCT
		reverse	ACGACACTCGAGGCTTTACG
	Mir200b -ve	forward	CTTGTCCCCCAAACACAGAT
		reverse	ACCCACATCTTCAGGCTGTC
	Mir205 -ve	forward	CTTTGCCACCTGCTAACTCC
		reverse	GGTCTGGGTAGGCACACATC
	Esrp1 -ve	forward	AGGGTAGGGGGCAGAGTAAA
		reverse	GGACGACCCCTTTACTGACA
	Esrp2 -ve	forward	GGCAGAGACCAAGTGATCTACC
		reverse	CCTTCCTCTCCCTCCCACT
	Ovol1 -ve	forward	AAATGTTTGCCGGAACAGAC
		reverse	TGAACCGTATCCACCTGTGA
	Ovol2 -ve	forward	CCAGCATCTTAAGCGGATCT
		reverse	TCTTCAATGGTTCCCAAAGC
	Zeb1 -ve	forward	AAGGCATGTTTATGGTATGTTATCTC
		reverse	CCCCTTTTCAAAGGAGGAAT

Disease Models & Mechanisms • Supplementary information
